# GPhenoVision: A Ground Mobile System with Multi-modal Imaging for Field-Based High Throughput Phenotyping of Cotton

**DOI:** 10.1038/s41598-018-19142-2

**Published:** 2018-01-19

**Authors:** Yu Jiang, Changying Li, Jon S. Robertson, Shangpeng Sun, Rui Xu, Andrew H. Paterson

**Affiliations:** 1 School of Electrical and Computer Engineering, University of Georgia, Athens, Georgia 30602 United States of America; 2 College of Agricultural and Environmental Sciences, University of Georgia, Athens, Georgia 30602 United States of America; 3 Franklin College of Arts and Sciences, University of Georgia, Athens, Georgia 30602 United States of America

## Abstract

Imaging sensors can extend phenotyping capability, but they require a system to handle high-volume data. The overall goal of this study was to develop and evaluate a field-based high throughput phenotyping system accommodating high-resolution imagers. The system consisted of a high-clearance tractor and sensing and electrical systems. The sensing system was based on a distributed structure, integrating environmental sensors, real-time kinematic GPS, and multiple imaging sensors including RGB-D, thermal, and hyperspectral cameras. Custom software was developed with a multilayered architecture for system control and data collection. The system was evaluated by scanning a cotton field with 23 genotypes for quantification of canopy growth and development. A data processing pipeline was developed to extract phenotypes at the canopy level, including height, width, projected leaf area, and volume from RGB-D data and temperature from thermal images. Growth rates of morphological traits were accordingly calculated. The traits had strong correlations (*r* = 0.54–0.74) with fiber yield and good broad sense heritability (*H*^2^ = 0.27–0.72), suggesting the potential for conducting quantitative genetic analysis and contributing to yield prediction models. The developed system is a useful tool for a wide range of breeding/genetic, agronomic/physiological, and economic studies.

## Introduction

Agriculture is facing tremendous challenges from the rapidly growing population that demands more food, feed, fiber, and fuel, as well as from the changing climate and severe shortfall of arable land and water resources^1^. To overcome these challenges, it is necessary to select and cultivate new crop genotypes with high yield and quality while using a reduced amount of natural resources such as water. Cotton (*Gossypium*) is the most important source of natural fiber, and in recent years it has also become an important source of food and feed (e.g. cottonseed oil for humans and hulls for livestock)^2^. Consequently, improving cotton production and quality is crucial to fulfilling the fiber and food requirement of over nine billion people by 2050^3^. Genetic/genomic research and breeding programs hold great potential to double the current production of cotton. Two key factors have been recognized for these programs: development of diversity panels and evaluation of phenotypic traits^4^. During past decades, advances in genetic technologies paved the way for genetic analysis of large crop populations. In particular, high-throughput sequencing techniques have enabled rapid and inexpensive genotyping of crop plants to provide thousands of recombinant lines for genetic selection and genomics studies. However, current phenotyping primarily relies on manual measurements and observations, and is far behind genotyping in terms of throughput, accuracy, and repeatability. This limits the potential use of crop genotypes in genotype-phenotype mapping and characterizing genotype-environment interactions^5^. High throughput phenotyping (HTP) has been recognized as an essential part of a new ‘Green Revolution’ to further improve crop yield and quality as well as to better understand crop genomics^5^.

In the past decade, greenhouse- and chamber-based high throughput phenotyping systems have been developed by several transnational companies, public institutions, and universities^6^. These systems are fully automated and can accurately measure phenotypic traits of individual plants. Many studies have demonstrated successful use of HTP systems to reveal relationships between genotypes and phenotypes^7–10^. These studies primarily focused on crop resistance and/or tolerance to stress and nutrient-deficiency, and the experiments took place in greenhouses, where environments were artificially controlled to simultaneously provide consistent ambient condition (e.g. illumination) for data collection and minimize confounding effects (spatial heterogeneity) on experiments. In addition, automation subsystems could efficiently and precisely implement treatments (e.g. irrigation and nutrient spray) for experiments, and provide accurate environment and treatment records (irrigated water and nutrient amount) for data analyses. Although the greenhouse-based HTP systems provide the aforementioned beneficial features, they have three major limitations. First, system development and maintenance are usually prohibitively expensive for small breeding programs. Second, most greenhouse- and chamber-based systems can only handle up to several hundred potted plants (or plant wells) in an experiment, so the maximum population size is far too small for breeding programs. Third, and most importantly, quantitative trait loci or candidate genes identified in controlled environments may be less effective in field conditions^4^. Plant growth and development can be significantly affected by environmental factors such as soil, and these factors are extremely difficult (or sometimes impossible) to simulate in controlled environments.

To address these issues, it is imperative to develop systems for field-based high throughput phenotyping (FB-HTP). In consideration of system cost, utility, and spatial and temporal resolution, FB-HTP systems based on high-clearance ground vehicles are currently preferred for breeding programs of moderate scale (up to a few thousand plots)^11–13^. Several representative systems have been developed recently^14–20^. Some of these systems^14–16^ have demonstrated usefulness in breeding programs and genomics studies^21–23^. It is noteworthy that imaging techniques were frequently applied in recent systems due to their ample capacity for extracting complex traits. The imaging techniques included conventional RGB, thermal, spectral, and 3D imaging modules. Integration of multimodal imaging sensors would significantly improve the sensing capability of an FB-HTP system, because a multimodal imaging system cannot only measure traits from a single imaging module, but also provide a set of traits from different imaging modules. For instance, RGB and 3D imagers can be integrated to provide both color and position information for plants or plant organs. Generally, the data volume of imaging modules is high, thus presenting a technical challenge in data acquisition. In particular, the continuous scanning mode is preferable for high throughput data collection in the field, which further increases the demand in a data acquisition (DAQ) system dedicated for field-based phenotyping systems. There are three features that need to be included in the DAQ system: high-throughput, customizability, and modularity. High-throughput means that the DAQ system needs to handle high-volume data generated by imaging sensors. Customizability allows researchers to quickly develop and upgrade an integrative FB-HTP with various imaging modules for different phenotyping purposes. Modularity splits individual modules into independent working environments, which prevents entire-system malfunction and reduces the amount of effort required in development, when adding or removing imaging modules.

The overall goal of the research described in this paper was to develop and evaluate a modular and customizable ground mobile system using multiple imaging modalities for FB-HTP. Specific objectives were to: (1) develop a modular and customizable ground mobile system integrated with multiple high resolution imagery modules including RGB-D, thermal, and hyperspectral cameras, (2) calibrate and validate the sensing system, and (3) evaluate the usefulness of the FB-HTP system for breeding programs and genomics studies.

## Development and validation of the ‘GPhenoVision’ system

### Platform mechanical design and implementation

**Figure 1:**
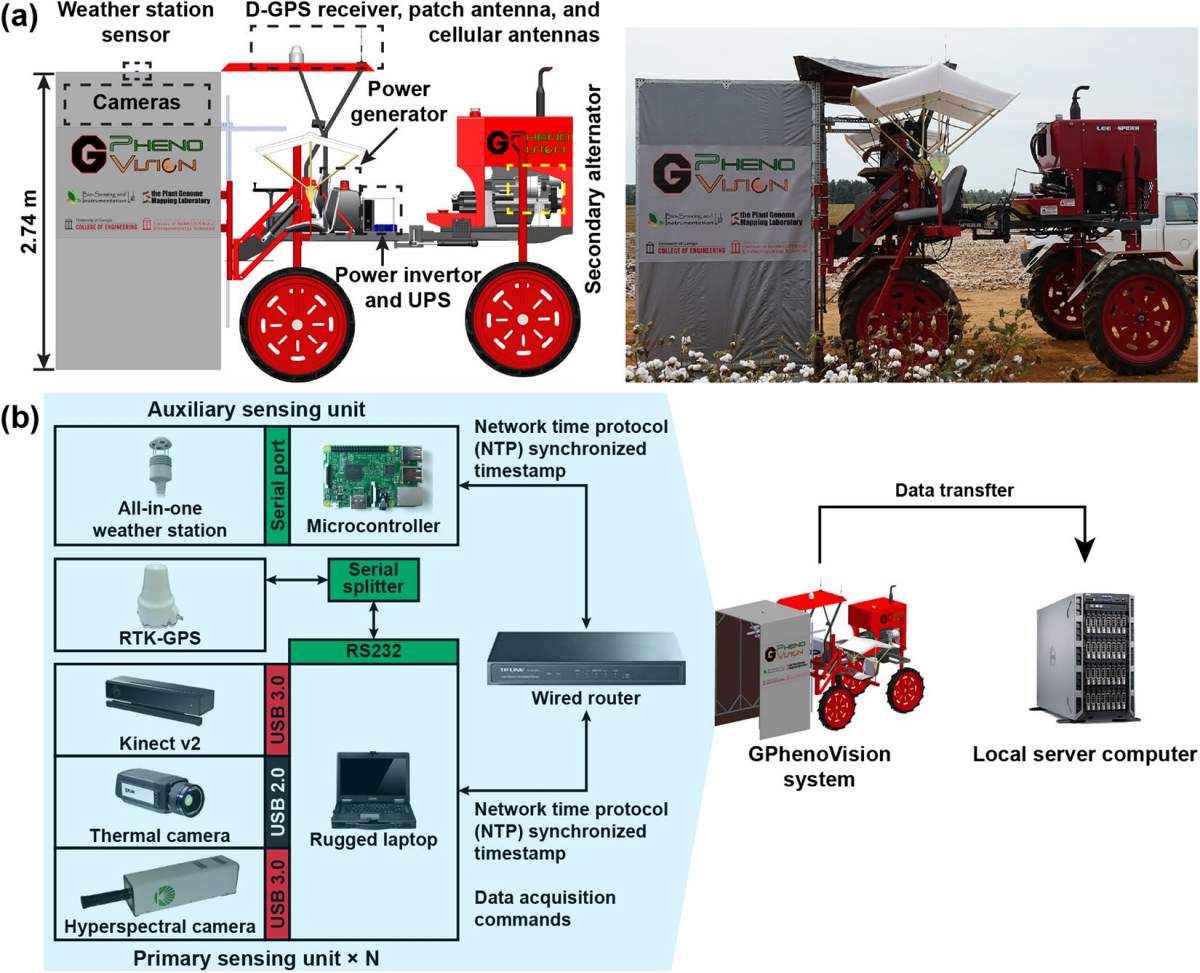
(**a**) Diagram and picture of the ‘GPhenoVision’ system design and (**b**) architecture of data flow and management. ‘N’ is the number of the primary sensing unit(s) integrated to the GPhenoVision system; N = 1 in the current study.

The ‘GPhenoVision’ system consists of five components including a platform, mechanical structures, sensing and electrical systems, and data acquisition software. A high-clearance tractor (Spider DP, LeeAgra Inc. Lubbock, TX, USA) was the platform upon which other system components were integrated (Fig. 1(a)). In the current study, the wheel track and clearance of the platform were set as 1.83 m and 1.5 m, respectively, and narrow wheels were selected to minimize mechanical disturbance. An aluminum frame (width of 1.52 m) with three polyethylene tarpaulins covering the top and two sides was built as an enclosure to provide a shaded area. The platform engine and a secondary alternator were installed in the back of the platform by default, so the enclosure was attached to the front of the tractor to reduce engine-induced variations. The enclosure covered plants for imaging to reduce ambient interferences such as strong sunlight and wind effects. An adjustable sensor frame was welded in front of the tractor and covered by the enclosure, the height of which could vary from 1.2 m to 2.4 m above the ground. Imaging sensors were installed on the sensor frame in custom camera holders, and a rubber cushion was added between metal frames to further reduce high-frequency vibrations that could be potentially transferred to sensors. This vibration reduction helped to decrease the possibility of acquiring blurry images. The installation positions of sensors were predetermined so that acquired images could be geo-referenced. On top of the enclosure and tractor cab, there were two sensor bases on which environmental sensors and positioning devices were mounted. In the middle of the platform, a metal frame was fabricated to hold instruments of the electrical system. Passenger seats were installed on each side of the tractor for testing purposes. In the testing stage, additional operators were on the tractor to test the image acquisition software during field data collection.

### Sensing system

The sensing system contained two subsystems: a DAQ system and local computing resources (Fig. 1(b)). The DAQ system consisted of a primary sensing unit, a positioning unit, and an auxiliary sensing unit, and was integrated into the platform for data collection in field conditions (left block in Fig. 1(b)). In the primary sensing unit, an RGB-D camera (Kinect for Windows v2, Microsoft, Redmond, WA, USA), a thermal camera (A655sc, FLIR Systems Inc., Wilsonville, OR, USA), and a hyperspectral camera (MRC-923-001, Middleton Spectral Vision, Middleton, WI, USA) were connected to a rugged laptop (S400, Getac Technology Corporation, Taipei, Taiwan) that was equipped with a solid state drive (SSD; 850 Pro 1TB, Samsung Electronics, Suwon, South Korea). The laptop used an Intel i7-4712 MQ CPU with 8 GB of RAM and Windows 10 Pro operating system. A serial port (RS232) of the laptop was utilized to receive GPS data from the positioning unit based on a real time kinematic GPS (RTK-GPS; Cruizer II, Raven Industries Inc., Sioux Falls, SD, USA). Based on each camera’s field of view (FOV) and scanning mode, the sampling frequencies of the RGB-D and thermal cameras were 6 frames per second (FPS), whereas the hyperspectral cameras were 100 FPS. All cameras used global shutter to avoid the Wobble effect during data collection. Accordingly, data volume of the primary sensing unit was estimated (Table 1). In the auxiliary sensing unit, a single-board computer (Raspberry 3 Model B, Raspberry Pi Foundation, Cambridge, UK) was used to receive and save ambient information (air temperature, relative humidity, and pressure) by controlling a microcontroller (Arduino Uno, Arduino, Vancouver, BC, Canada) that regulated an environmental sensor (BME280, Bosch Sensortec, Gerlingen, Germany). The primary and auxiliary sensing units were wired to a router for communication of synchronized timestamps and DAQ commands.

**Table 1:** Key specification and data volume of the cameras used in the primary sensing unit.

	RGB-D camera	Thermal camera	Hyperspectral camera
Manufacturer	Microsoft	FLIR	Middleton Spectral Vision
Model	Kinect for Windows v2	A655sc	MRC-923-001
Scanning mode	Area scan	Area scan	Line scan
Field of view	70° × 60°	80° × 64.4°	55.9°
Data type	16-bit unsigned integer (depth) 32-bit unsigned integer (color)	32-bit single precision	16-bit unsigned integer
Spectral range	Visible (color), 827–850 nm (depth)	7.5–14 *μm*	400–1000 nm
Image resolution	512 × 424 (depth) 1920 × 1080 (color)	640 × 480	640(*spatial*) × 236(*spectral*)
Image size	0.42 MB (depth) 8 MB (color)	1.18 MB	0.29 MB
Designed frame rate (maximum)	6 (30) FPS (depth) 6 (30) FPS (color)	6 (30) FPS	100 (200) FPS
Data volume (maximum)	2.52 (12.6) MB/s (depth) 48 (240) MB/s (color)	7.08 (35.4) MB/s	29 (58) MB/s
Total data volume per second	86.6 (346) MB/s

**Table 2:** Summary of calibration and validation experiments for the RGB-D, thermal, and hyperspectral cameras used in the GPhenoVision system.

Sensor	Test parameter	Test location	Test objective
RGB-D camera	Depth accuracy	Field	Validate the accuracy of sensor measurements of morphological traits
Thermal camera	Measurement accuracy (compared with thermocouple measurements) for an object with various temperatures	Laboratory	Validate the measurement accuracy in a control environment to provide an accuracy baseline
Thermal camera	Measurement accuracy (compared with thermocouple measurements) for an object at different distance to the camera	Field	Validate the repeatability of measurements due to the change of object-to-camera distance
Thermal camera	Temperature differences between thermal camera measurements for plants under shaded and unshaded areas	Field	Verify the absence of the shading effect to canopy temperature measurements
Hyperspectral camera	Accuracy of the regression model between pixel locations on the spectral dimension and standard wavelengths	Laboratory	Calibrate and validate the relationship between pixel locations on the spectral dimension and wavelengths in a scan line
Hyperspectral camera	Maximum and minimum spatial resolution	Field	Calibrate and validate the spatial resolution of the camera under various object-to-camera distances

After field data collections, data could be transferred to local and remote computing resources for data processing and sharing (right block in Fig. 1(b)). A server computer was equipped with a redundant array of independent disks (RAID 1) of 4 terabytes for storing data generated in one growth season. The server computer was also used to perform algorithms for extracting phenotypic traits.

### Electrical system

The electrical system was based on the secondary alternator (200 amps) of the tractor platform, an absorbed glass mat (AGM) battery (Yellowtop D34, OPTIMA Batteries, Inc., Milwaukee, WI, USA), a power inverter (APS1000-12, Power Bright, Fort Lauderdale, FL, USA), and an uninterruptible power supply (UPS; Back-UPS Pro 1500, APC by Schneider Electric, West Kingston, RI, USA). When the system ran, the alternator continuously generated and output power to the power inverter through the battery. The inverter boosted the input power from 12-volt direct-current (DC) to 120-volt alternating current (AC) and transferred to the UPS to provide stable AC output for the sensing system. A 2-kilowatt power generator (EU2000i, Honda Power Equipment, Alpharetta, GA, USA) was chosen as an alternative power generating source in case the secondary alternator malfunctioned. In total, the electrical system using either the secondary alternator or the power generator could provide AC output of 865 watts with the UPS protection, which was adequate to power the sensing system used in GPhenoVision (see Supplementary Table S1).

### Data acquisition software

**Figure 2:**
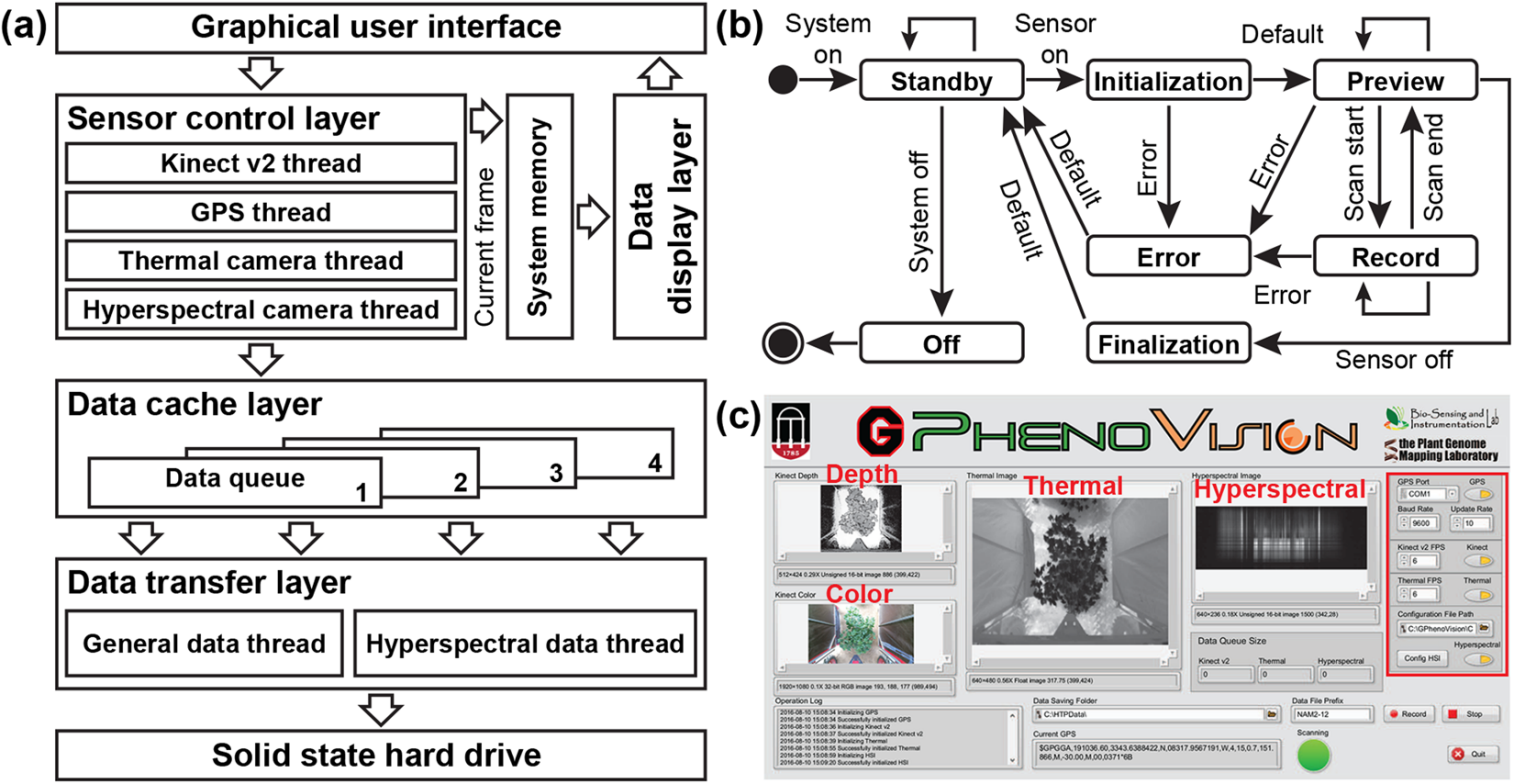
Design of the data acquisition (DAQ) software for the GPhenoVision system: (**a**) software architecture of the DAQ program; (**b**) the event-driven finite state machine (EFSM) developed for sensor threads in the sensor control layer; and (**c**) the front panel of the custom DAQ program.

Custom computer software was developed using LabVIEW 2015 (National Instruments, Austin, TX, USA) and deployed on the rugged laptop to control the positioning and primary sensing units for data acquisition. The software was based on a multilayered architecture; each layer worked independently but the layers could be synchronized with user commands (Fig. 2(a)). The graphical user interface (GUI) received user commands and transferred them to the sensor control layer for processing. The GUI also worked with the data display layer to present current image frames to users. The sensor control layer utilized multiple threads, with each thread controlling a single sensor through an event-driven finite state machine (EFSM) (Fig. 2(b)). Due to the multithread design, the GPS and the RGB-D, thermal, and hyperspectral cameras could acquire data using different sampling frequencies. Additionally, although all sensors shared the same EFSM, sensor initialization, finalization, image preview, and acquisition relied on specific drivers or software development kits (SDKs) for each sensor. In the present study, the RGB-D camera required its driver to be provided by the manufacturer and a third-party LabVIEW library (Haro3D, HaroTek LLC, Keller, TX, USA), whereas the thermal and spectral cameras required only their official SDKs. The serial communication virtual instruments (VIs) provided by LabVIEW were used to receive data from the GPS. As the total data volume could be up to 346 MB/s, it was necessary to design a data cache layer, which increased the performance of data transferring and storage by buffering data from sensors in the computer memory. The data transfer layer periodically inquires the data cache layer and writes buffered data back to the SSD hard drive.

The software front panel showed data (GPS, depth, color, thermal, and hyperspectral images), memory cache status, and operation log in real time during field data collection (Fig. 2(c)). This allowed operators to quickly read height or temperature of plants from raw images or investigate potential issues such as failure of certain sensors. Sensors could be individually configured and controlled through sensor control panels (indicated with red rectangle in Fig. 2(c)). The collected images were saved in a folder specified by the operator. After configuring and turning on certain sensors, an operator could start and stop data collections by using the ‘Record’ and ‘Stop’ buttons, accordingly.

In addition to the aforementioned computer software, a custom Python (Python 2.7) script was developed and deployed on the single-board computer to acquire air temperature, relative humidity, and pressure with acquisition timestamps under 1 Hz. Timestamps of the primary and auxiliary sensing units were synchronized through a network time protocol (NTP) service that was installed on the rugged laptop. Due to its small amount of data (35 bytes per second), the auxiliary sensing unit was controlled to start/stop by a physical button, and continuously recorded data during a whole data collection session without being interrupted by commands from the DAQ software controlling the primary sensing unit.

### System calibration and validation

#### Calibration and validation methods

Prior to using the system, it was important to calibrate and validate sensors in the primary sensing unit because they would provide essential data for extracting phenotypic traits. The three cameras were used to acquire images for different types of traits, and thus they had various aspects to be calibrated or validated (see Table 2 for detailed information for calibration and validation). For the Kinect v2 camera, depth accuracy is a key factor because it determines the accuracy of each point in a colored point cloud retrieved from the sensor. Although the depth accuracy of Kinect v2 camera has been proven in both laboratory and field conditions^24,25^, it was necessary to re-validate sensor measurements as the camera was integrated into a new system. To account for ambient effects such as winds, depth accuracy of the Kinect v2 camera was evaluated by measuring plant height in field conditions. The GPhenoVision system scanned 100 cotton plants on three different days while recording wind speed information with a portable anemometer (HYELEC MS6252B, Huayi Electronics Industrial Ltd., Hangzhou, Zhejiang, China). Plant heights were extracted from depth images by using the method described in a previous study^25^, and also manually measured on each day for reference. The system and manual measurements were compared using simple linear regressions, and the adjusted *R*^2^ and root mean squared error (RMSE) were used as accuracy indicators.

Three experiments were conducted to validate measurement accuracy of the thermal camera under various conditions. The first experiment was to provide a baseline of the thermal camera accuracy by measuring surface temperature of a blackbody (an object with emissivity of 1; 4-inch Blackbody Source, FLIR Systems Inc., Wilsonville, OR, USA) in laboratory conditions. The thermal camera and blackbody were placed on the same plane separated by a distance of 2.4 m (the maximum distance between objects and the thermal camera on the system), and a T-type thermocouple was attached to a corner of the blackbody surface to measure its temperature for reference. The camera and thermocouple measured temperatures synchronously. A total of 26 surface temperatures ranging from 24 °C to 69 °C were set to the blackbody by adjusting its powering voltage, and each temperature was measured by both the thermal camera and thermocouple for 200 frames; therefore, both the thermal camera and thermocouple were used to collect 200 data points. The mean value of the 200 data points collected by each sensor was calculated as the sensor measurement at each known blackbody temperature. The mean values obtained from the thermal camera and thermocouple were compared using linear regressions, and the adjusted *R*^2^ and RMSE values were used for accuracy evaluation.

Although the first experiment provided the baseline of thermal camera accuracy, the baseline was obtained in laboratory conditions that were different from field conditions (practical data collection conditions). The second experiment was to test whether the thermal camera could repeatedly provide accurate measurements of objects at different distances from the camera in field conditions. The thermal camera was oriented towards nadir, installed on the GPhenoVision system, and raised to the highest position (2.4 m above the ground). The blackbody was set to a constant voltage (therefore a constant surface temperature in theory), and placed on a tripod that was in the center of the camera’s FOV. The blackbody was raised from 0 to 1.5 m above the ground with an interval of 0.1 m, and at each height, its surface temperature was measured for 200 frames (therefore yielding 200 data points) by both the thermal camera and thermocouple. Since blackbody temperature could vary due to fluctuations of ambient conditions, the thermal camera measurements were compensated by using Equation 1. Analysis of variance (ANOVA) test was conducted on the compensated measurements to identify any existence of statistical differences among temperatures measured at different heights.

1 $$T{I}_{h,i}^{compensated}=T{I}_{h,i}^{raw}+T{C}_{h,i}-T{C}_{h,i},\quad h=0,0.1,...,1.5,\quad i=1,2,...,200$$

where *TI* and *TC* represented temperatures measured by the thermal camera and thermocouple, respectively; *h* indicated heights of blackbody; and *i* was the number of frames (or data points) at a certain height.

During data collection, plants would be shaded by the enclosure and this might raise concern regarding shading effects on temperature measurements. The last experiment was to test any shading effect on measurements of canopy temperature in field conditions. In the experiment, only half of the enclosure was covered so that the thermal camera could acquire images of plants under both shaded and unshaded conditions. The GPhenoVision system with the modified enclosure was used to scan 30 plants on three days (14 July 2016, 22 July 2016, and 26 July 2016). A total of 90 pairs of images were collected, each containing the same single plant that appeared in both the shaded and unshaded areas under the modified enclosure. The plant in each image was masked by a thresholding method to calculate its mean canopy temperature. Differences in temperatures measured under the two shading conditions were computed, and the mean value and standard deviation of the temperature differences were used to evaluate shading effects. During data collection, shading would be considered to have no effect on canopy temperature measurements if a mean difference of zero was achieved with a standard deviation of less than 0.5 °C.

The hyperspectral camera was calibrated in spectral and spatial dimensions, respectively. A method proposed in a previous study^26^ was used to calibrate the spectral dimension. To obtain a more accurate regression, three calibration lamps were used including a Krypton lamp (Model 6031, Oriel Instruments, Stratford, CT, USA), a Xenon lamp (Model 6033, Oriel Instruments, Stratford, CT, USA), and an Hg (Ar) lamp (Model 6035, Oriel Instruments, Stratford, CT, USA). Additionally, the spatial resolution was calculated and tested using two test targets that were printed on letter-size (215.9 mm × 279.4 mm) bright white papers. The first target contained two resolution patterns of 1/8 and 1/6 line pairs per millimeter (LP/mm) with each of 15 pairs, and the second target contained three resolution patterns of 1/20, 1/15, and 1/10 LP/mm with each of 5 pairs. The targets were placed at three heights to evaluate spatial resolutions under the worst (targets were on the ground), common (targets were 1 m above the ground), and best (targets were 1.5 m above the ground) conditions. The spatial resolution under each condition was calculated as a ratio of the target length (279.4 mm) to the number of pixels representing the target in an image. Subsequently, the calculated spatial resolutions were validated by using the resolution patterns. If a resolution pattern was correctly identified in an image, two image pixels at least should be assigned to any line pair in the pattern with one pixel for the black stripe and another for the white. As a result, the spatial resolution was confirmed by the resolution patterns using Equation 2.

2 $$(\begin{array}{cc}SR\le \frac{1}{2\times r}, & {\rm{if}}\,{\rm{pattern}}\,{\rm{was}}\,{\rm{correctly}}\,{\rm{identified}}\\ SR > \frac{1}{2\times r}, & {\rm{otherwise}}\end{array}$$

where *SR* is the camera spatial resolution in mm/pixel and *r* is the pattern resolution in LP/mm.

#### Calibration and validation results

**Figure 3:**
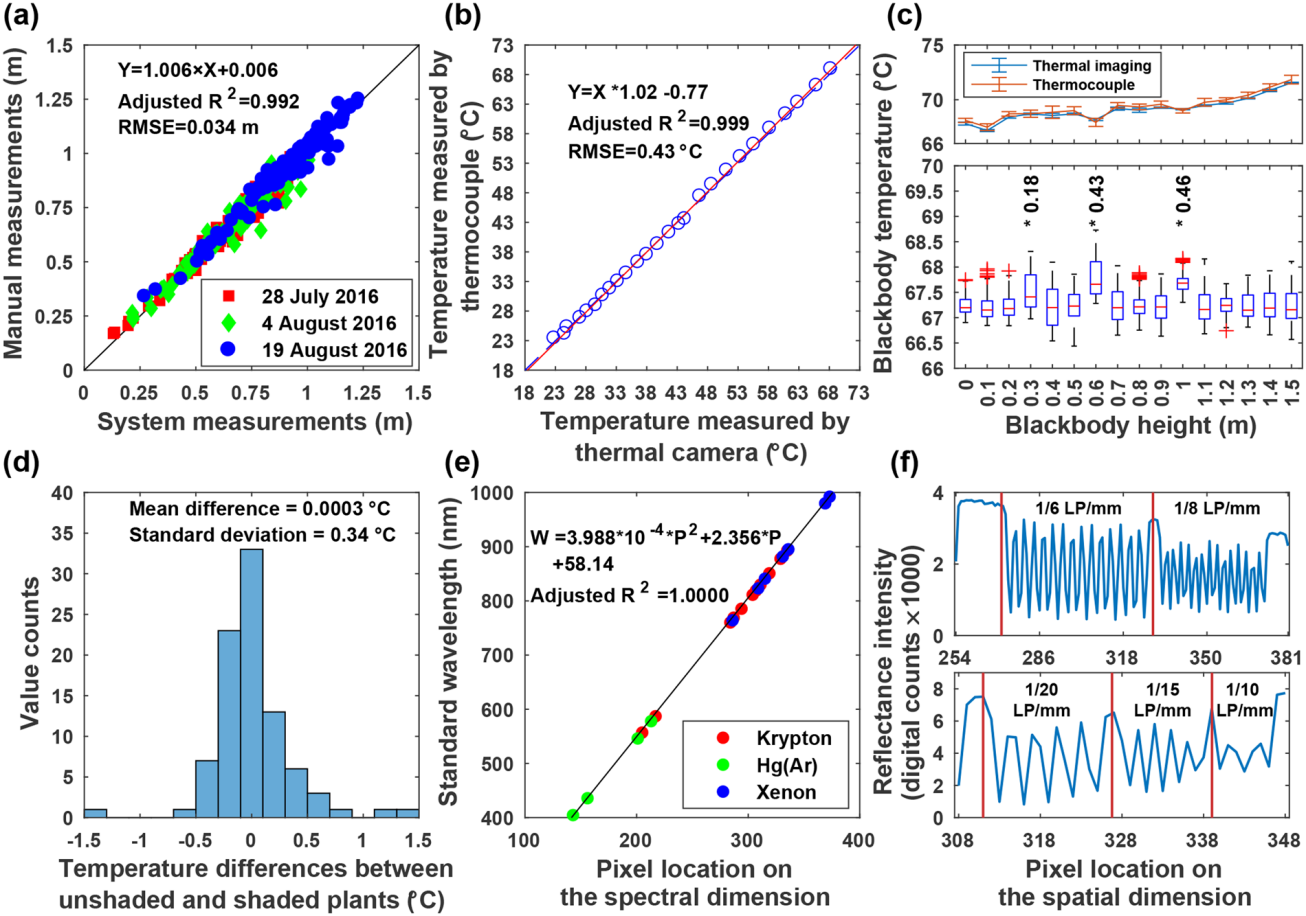
Calibration and validation results of three sensors used in the primary sensing unit. (**a**) Depth accuracy of the RGB-D camera. (**b**) Regression between blackbody temperatures measured using thermal camera and thermocouple in laboratory conditions. (**c**) Raw (top) and compensated (bottom) blackbody temperatures measured using thermal camera in field conditions when placing blackbody at various heights in the sensing range. (**d**) Canopy temperatures measured under shaded and unshaded conditions. (**e**) Regression between wavelengths and pixel locations on the spectral dimension in images. (**f**) Spatial resolution of the hyperspectral camera under the best (top) and worst (bottom) situations.

Overall, the system and manual measurements of plant height were strongly correlated (*adjusted R*^2^ 0.99) with an RMSE of 0.034 m for data on all three days (Fig. 3(a)). Compared with results from another study^25^, both *adjustedR*^2^ and RMSE were slightly improved, indicating that the GPhenoVision system could accurately measure plant height in field conditions. Additionally, these results were stably obtained when using data on individual days (see Supplementary Table S2), which meant that the system had high repeatability to accurately measure plant height under various conditions.

For the blackbody surface temperature, thermal camera and thermocouple measurements were highly correlated, with an RMSE of 0.43 °C, and thus thermal camera measurements were as accurate as traditional contact measurement methods (Fig. 3(b)). Consequently, thermal images acquired by the camera could be directly used for extracting canopy temperature by configuring a proper emissivity. The emissivity can be set from 0.93 to 0.99 depending on crops to be studied^27^. A value of 0.96 is commonly used as cotton plant emissivity^28^ and has been used for field data collection in the present study. In the second experiment, although the blackbody was set at a constant voltage, its surface temperatures measured by raw thermal images and thermocouple varied when the blackbody was placed at different heights (top chart in Fig. 3(c)). This indicated that the temperature variations were primarily due to fluctuations of ambient conditions. After compensation, thermal imaging measurements showed no statistical difference with thermocouple measurements when the blackbody was placed at various heights (bottom chart in Fig. 3(b)). Exceptions occurred at the heights of 0.3, 0.6, and 1 m above the ground, but the temperature differences at these three heights were less than the nominal camera accuracy (0.5 °C). As a result, temperature measurements with the thermal camera were consistently accurate as long as plants were within the system measurement range (0 to 1.5 m). The mean difference in canopy temperatures between plants under shaded and unshaded areas was negligible, because the actual difference (3 × 10^−4^ °C) was smaller than the nominal camera sensitivity (0.05 °C). Additionally, the standard deviation was less than the camera’s nominal accuracy, which meant that there was no shading effect on canopy temperature measurements (Fig. 1(d)). This was probably because canopy temperature would not immediately decrease when plants were shaded for only one or two seconds during data collection. Therefore, images acquired by the thermal camera of the GPhenoVision system could be used to accurately extract physiological traits such as canopy temperature.

Following a previously developed method^26^, no “keystone” or “smile” distortion was observed in the spatial and spectral dimensions (see Supplementary Figure S1), and thus correction of distortion was not needed for the hyperspectral camera. In the spectral calibration, 22 representative wavelengths from 404.66 nm to 992.3 nm were identified and used to establish the regression equation between pixel locations on the spectral dimension and wavelengths (Fig. 3(e)). The regression equation was satisfactory because the standard and calculated wavelengths were strongly correlated (*adjusted R*^2^ = 1). The RMSE (less than 1 nm) was also acceptable because the spectrograph has a nominal spectral resolution of 2.7 nm. In the spatial calibration, the length (279.4 mm) of the two targets was recognized as 41, 74, and 128 pixels in hyperspectral images acquired at the worst (on the ground), common (1 m above the ground), and best (1.5 m above the ground) conditions. Thus, the spatial resolutions of the hyperspectral camera ranged from 2.2 to 6.8 mm/pixel and was 3.8 mm/pixel under the common condition. The worst and best resolutions were validated by the test patterns on the targets. In the worst condition, resolutions of lower than 1/15 LP/mm were successfully identified as 5 pairs, whereas resolutions of 1/10 LP/mm were not correctly recognized (bottom chart in Fig. 3(f)). Based on Equation 2, this confirmed that the spatial resolution (6.8 mm/pixel) was better than 7.5 mm/pixel but worse than 5 mm/pixel. The hyperspectral camera successfully resolved all patterns in the best condition, which agreed with the calculated spatial resolution of 2.2 mm/pixel (better than 3 mm/pixel; top chart in Fig. 3(f)). Nonetheless, even the worst spatial resolution would be 6.8 mm/pixel, which was smaller than the size of a cotton leaf. Thus, the hyperspectral camera of the GPhenoVision system has an adequate spatial resolution for many elements of phenotyping.

## Results

### Representative images acquired by the system

**Figure 4:**
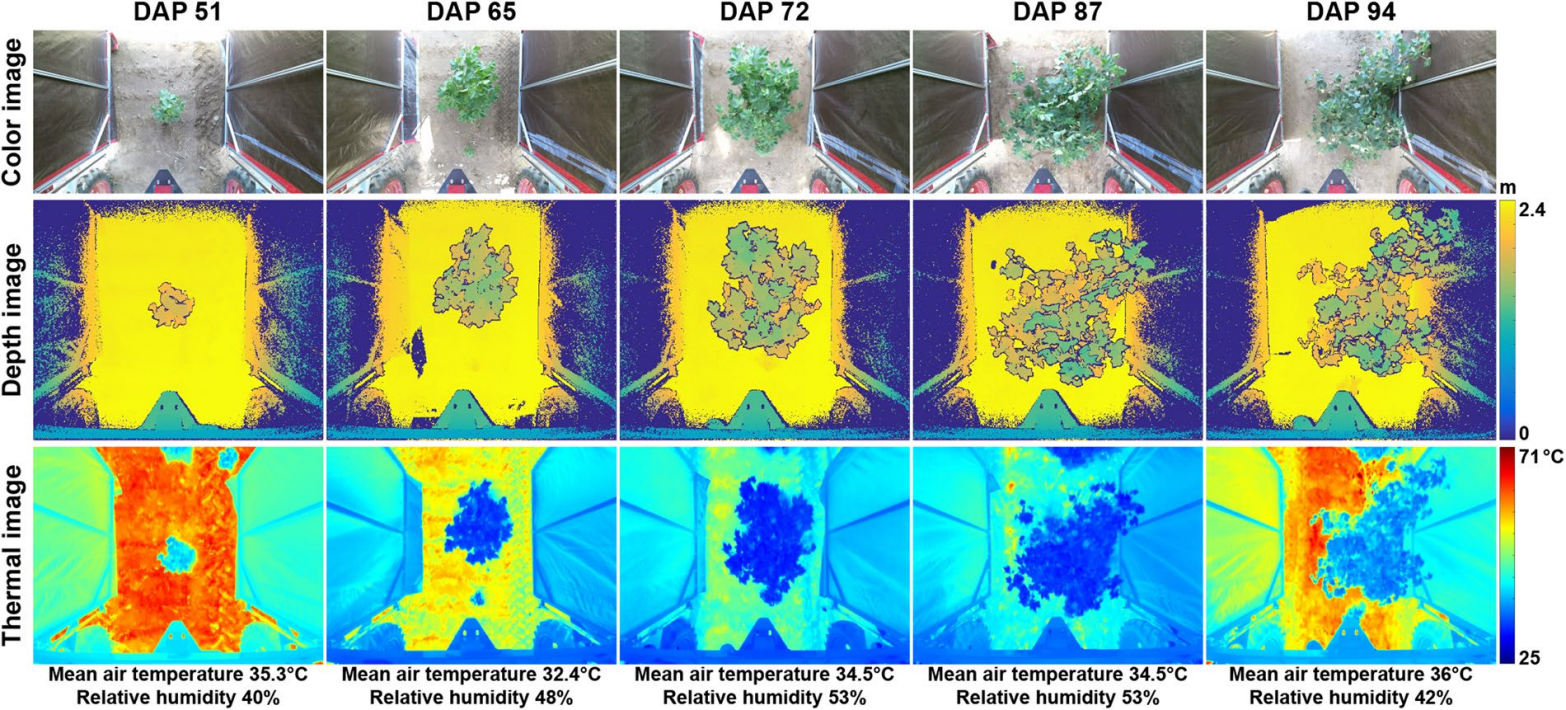
Representative color, depth, and thermal images acquired by the GPhenoVision system on five days in the field.

The GPhenoVision system could successfully control the RGB-D and thermal cameras to acquire depth, color, and thermal images of cotton plants under field conditions (Fig. 4). The system demonstrated the capability of simultaneously acquiring and storing high-volume images generated by multiple imaging sensors of high resolution. Therefore, the system could be a useful tool for field data collection, and the system design is reusable for FB-HTP projects aiming to utilize high-resolution imaging sensors.

Additionally, the acquired images could potentially be used to extract various phenotypic traits.. The depth images contained 3D information about plant canopies, which was useful to extract morphological traits such as canopy height, projection area, and volume. In combination with color images, depth images could be converted to colored point clouds in which vegetative components were easily segmented from the background. This might improve accuracies of measuring morphological traits, compared with other 3D imaging sensors such as LiDAR. Furthermore, color features were important for detecting certain plant organs such as flowers. Thermal images had an advantage in measuring not only canopy temperatures but also the temperature distribution over a canopy. Some leaves were cooler than others, probably due to their geometric distributions or physiological responses to environments.

### Extracted phenotypic traits

**Figure 5:**
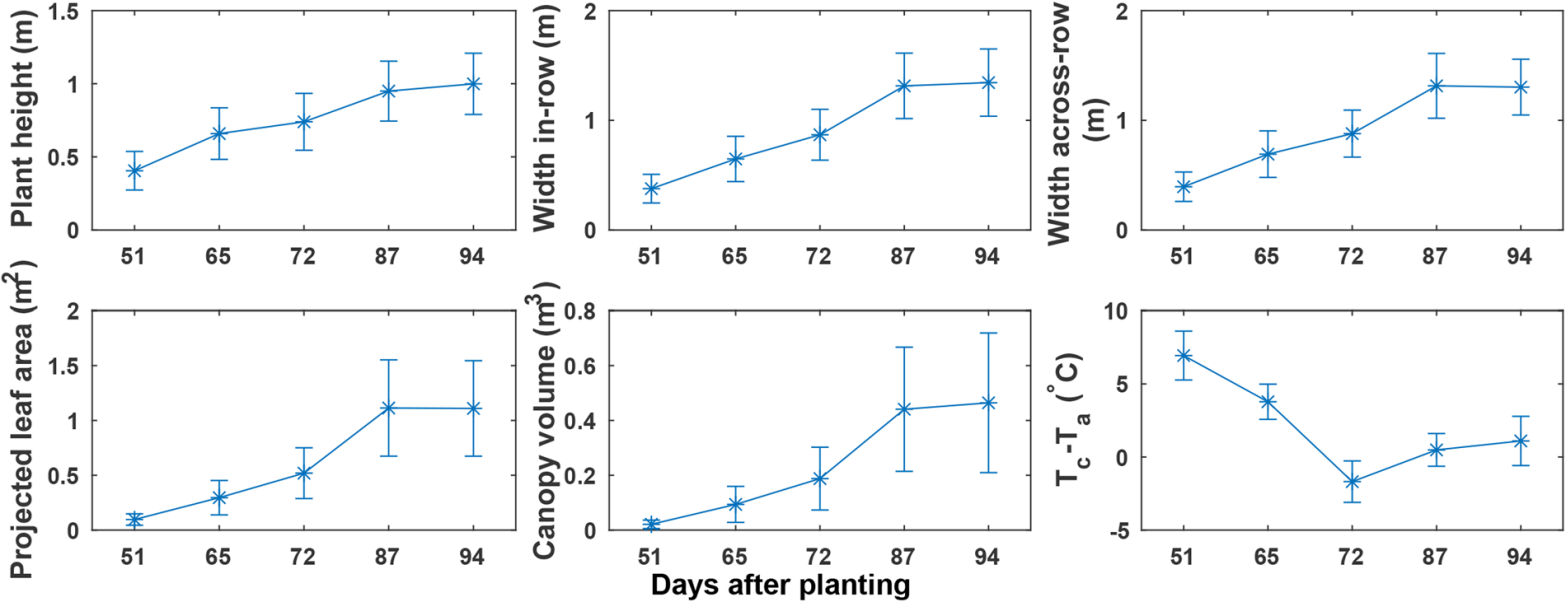
Six phenotypic traits measured using the GPhenoVision system on 14 July (51 days after plating, DAP 51), 28 July (DAP 65), 04 August (DAP 72), 19 August (DAP 87), and 26 August (DAP 94) in 2016. Asterisk and error bar indicated the mean value and standard deviation of traits for all plants in the field, respectively. DAP 51, 65, and 72 were in canopy development stage, and DAP 87 and 94 were in flower and boll development stage.

Overall, plant canopies elongated and expanded substantially from DAP (day after planting) 51 to 87 (canopy development stage), but only slightly after DAP 87 by which time the flower and boll development stage had started (plant height, width in-row (WIR), and width across-row (WAR) in Fig. 5). Projected leaf area (PLA) followed the trends of plant width, whereas canopy volume (CV) followed the trends of both plant height and width (projected leaf area and canopy volume in Fig. 5). This was because projected leaf area was primarily affected by plant expansion, but canopy volume was affected by both canopy elongation and expansion. Thus, any single dimensional development led to increases of canopy volume. Canopy temperature showed a different trend than canopy expansion (Tc-Ta in Fig. 5). Before DAP 72, canopy temperatures decreased, as plants rapidly developed and increased leaf area available for transpiration (resulting in evaporative cooling). The decrease of canopy temperature was also due to environmental factors. The total precipitation levels were 111.76 mm and 178.82 mm during the period from the day of planting to DAP 51, and from DAP 66 to 72, respectively, resulting in an increase of average daily precipitation from 2.23 mm to 25.55 mm (see Supplementary Table S3 for detailed precipitation information). However, from DAP 73 to 94, environmental factors became dominant. Average daily precipitation was 6.21 and 3.19 mm per day from DAP 73 to DAP 84 and DAP 85 to 94, respectively. Reduced water availability may reduce transpiration, leading to higher canopy temperatures that may indicate water stress. The plant height, projected leaf area, and canopy temperature showed similar trends with previous studies^15,16^. Moreover, GPhenoVision could measure multi-dimensional phenotypic traits such as projected leaf area and canopy volume, providing new tools for understanding canopy growth and development.

### Correlation between extracted traits and fiber yield

Morphological traits generally had clear positive correlations (*r* 0.5) with fiber yield (Table 3, see Supplementary Figure S2.1–S2.5 and Table S4.1–4.5 for detailed results). The correlations became strong until DAP 87 and then were relatively weak on DAP 94. This is consistent with findings that canopy growth and development primarily contributes to fiber yield in early stages rather than late stages^29^. Single-dimensional traits were comparable (sometimes slightly better) with 2D and 3D traits in terms of correlation with fiber yield. This was likely due to the single plant layout where a plot contained only one plant. Compared with usual plot design, plant height in the single plant layout could be more indicative of plant growth and development, showing strong correlation with fiber yield. High canopy temperature, an indicator of plant water stress, mostly showed negative correlations with fiber yield. However, no correlation was observed between fiber yield and canopy temperatures measured on DAP 72, when ample water was available, suggesting that at that time growth was not constrained by water limitation and thus canopy temperature was not predictive of fiber yield^16^.

**Table 3:** Pearson correlation coefficients between fiber yield and phenotypic traits measured on five days after planting (DAP) in 2016.

Date (DAP)	Plant height	Width in-row	Width across-row	Projected leaf area	Canopy volume	Tc-Ta
14 July (51)	0.65***	0.67***	0.70***	0.66***	0.59***	−0.65***
28 July (65)	0.68***	0.65***	0.61***	0.60***	0.49***	−0.44***
04 August (72)	0.68***	0.64***	0.66***	0.63***	0.62***	NS
19 August (87)	0.69***	0.64***	0.62***	0.66***	0.58***	−0.45***
26 August (94)	0.56***	0.60***	0.65***	0.60***	0.53***	−0.25*

Pearson correlation tests used data from 100 plants (n = 100) in the field. Asterisks (or abbreviations) indicated different statistical signifiance levels: NS for not significant, * for p-value < 0.05, ** for p-value < 0.01, *** for p-value < 0.001.

During the entire phenotyping period (DAP 51 to 94), growth rates of all morphological traits showed positive correlations with fiber yield (Table 4, see Supplementary Figures S3.1–S3.5 and Tables S5.1–5.5 for detailed results). Growth rates of projected leaf area and canopy volume had stronger correlations with fiber yield than plant heights and widths, suggesting that growth rates of 2D and 3D traits might be better predictors than those of 1D traits in a linear model for yield estimation. It was noteworthy that growth rates of WIR, WAR, PLA, and CV had no statistical correlation with fiber yield from DAP 87 to 94, because reproductive growth became dominant in this period and WIR, WAR, PLA, and CV did not change significantly. However, plant height increased slightly in that period, because excessive vegetative growth was from a single terminal bud located at the tallest part of branches. A moderate negative correlation was observed between fiber yield and growth rate of plant height from DAP 87 to 94, supporting the hypothesis that excessive vegetative growth may reduce reproductive growth in late stages of crop development. Therefore, a combination of growth rates of multi-dimensional morphological traits would be particularly useful for studying plant energy use efficiency in different growth stages.

**Table 4:** Pearson correlation coefficients between fiber yield and daily phenotype growth rates calculated between different days after planting (DAP) in 2016.

Period	Plant height	Width in-row	Width across-row	Projected leaf area	Canopy volume
14 July to 28 July (DAP 51-65)	0.54***	0.39***	0.32**	0.52***	0.43***
28 July to 04 August (DAP 65-72)	NS	NS	NS	0.51***	0.58***
04 August to 19 August (DAP 72-87)	NS	0.22**	0.21**	0.52***	0.38***
19 August to 26 August (DAP 87-94)	−0.36**	NS	NS	NS	NS
14 July to 26 August (DAP 51-94)	NS	0.34***	0.29**	0.55***	0.50***

Pearson correlation tests used data from 100 plants (n = 100) in the field. Asterisks (or abbreviations) indicated different statistical signifiance levels: NS for not significant, * for p-value < 0.05, ** for p-value < 0.01, *** for p-value < 0.001.

### Differences in extracted traits among genotype groups

**Figure 6:**
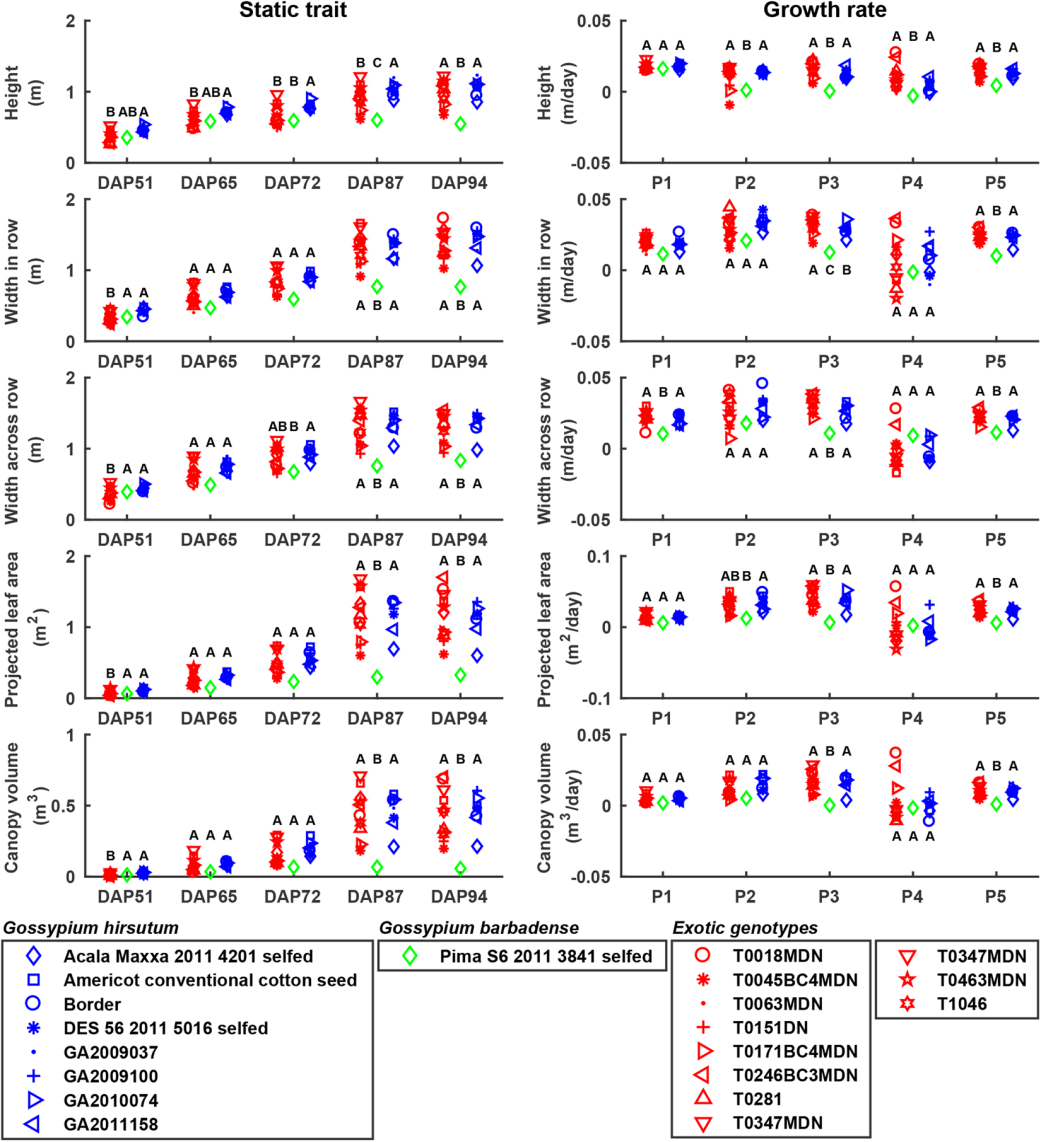
Differences in extracted traits among three cotton groups: *Gossypium hirsutum*, *Gossypium barbadense*, and ‘exotic’ genotypes. Growth rates were calculated in five periods including P1 (DAP 51-65), P2 (DAP 65-72), P3 (DAP 72-87), P4 (DAP 87-94), and P5 (DAP 51-97). Each marker indicated the mean value of traits for a genotype used in the study, and genotypes in the same group were rendered as the same color. Groups with different letters were statistically significant with each other (see Supplementary Table S6 for detailed p-values), and group mean values were sorted alphabetically. DAP 51, 65, and 72 (accordingly P1 to P3) were in canopy development stage, and DAP 87 and 94 (P4) were in flower and boll development stage.

Overall, *G. barbadense* had the lowest value of all traits after DAP 65, with a shorter and smaller plant canopy than *G. hirsutum* and “exotic”™ genotypes (Fig. 6). Plant height of *G. barbadense* Pima S6 reached its maximum on DAP 65, 22 days earlier than the *G. hirsutum* and “exotic” genotypes, whereas other static traits generally reached the highest level on DAP 87. These data might indicate abnormal vegetative growth of *G. barbadense* genotypes, which are not well adapted to the study area and not generally grown there. Manual field assessment showed all *G. barbadense* plants to be lodged, although most were otherwise healthy and continued to grow horizontally.

Plant canopy size (projected leaf area) of “exotic” genotypes was smaller than those of *G. hirsutum* and *G. barbadense* cultivars on DAP 51, indicating that the exotics established their maximum size slower than the two cultivated genotype groups. This was expected because the exotics tend to be late flowering and experience rapid vegetative growth in late stages. After DAP 51, the exotics did indeed experience rapid vegetative growth, consistently as high as those of *G. hirsutum* genotypes, resulting in large plant canopies that were comparable with *G. hirsutum* genotypes in late stages (after DAP 87). Some “xotic” genotypes (T0246BC3MDN, T0018MDN, T0347MDN, and T0151DN) had even larger canopies than *G. hirsutum* genotypes and maintained high vegetative growth rates, perhaps reflecting greater investment in vegetative growth and less in reproductive growth (seed and associated fiber).

Trait variations of the exotics were larger than those of elite *G. hirsutum* genotypes, especially static traits after DAP 87 and growth rates in the period from DAP 87 to DAP 94. This was expected, because elite *G. hirsutum* genotypes have been selected by plant breeders for many years and are generally very closely related to one another. On the other hand, the exotics harbor substantial genetic variations, some of which may be used to improve *G. hirsutum* genotypes by crossing and selection.

### Broad sense heritability of extracted traits

**Figure 7:**
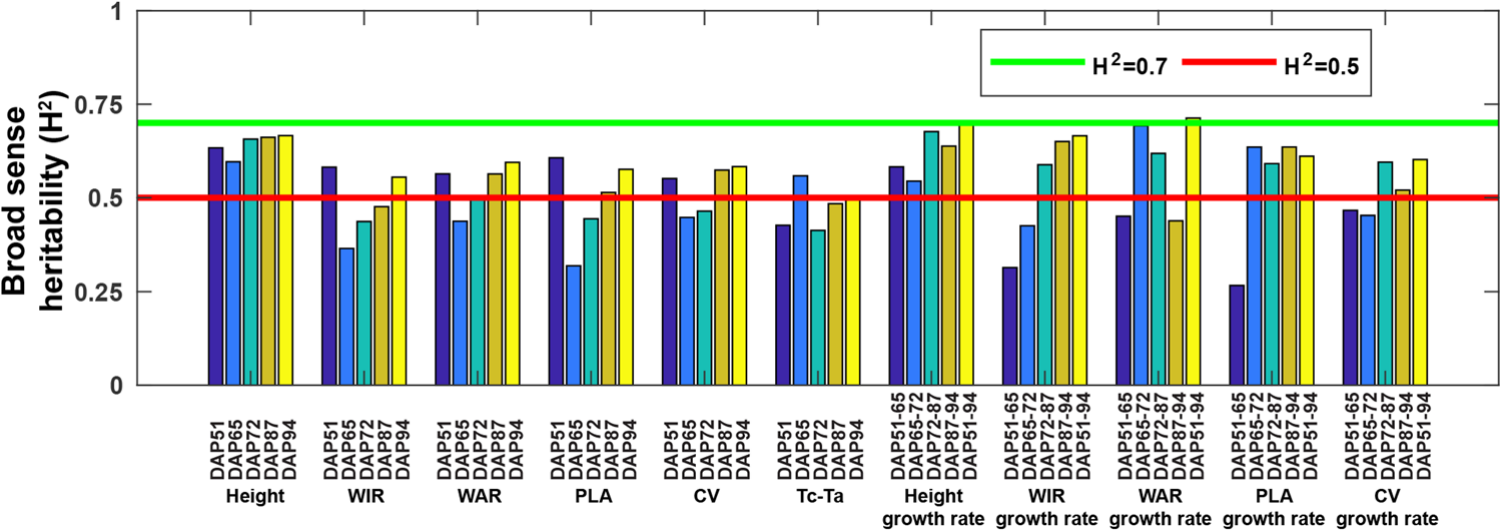
Broad sense heritability (*H*^2^) of phenotypic traits extracted in the present study. *H*^2^ 0.5 indicated a trait would be useful for genotype selection, and *H*^2^ 0.7 indicated a trait would be useful for genotype differentiation.

Most of the measured traits had broad sense heritability (*H*^2^) greater than 0.5, supporting the usefulness of the GPhenoVision system in genomics/genetics studies. *H*^2^ values of morphological traits (static traits) decreased to their lowest values in the middle of the growing season, and then began to increase and ultimately reached the maximum values at the last measurement date (Fig. 7). In particular, *H*^2^ values for all morphological traits were larger than 0.5 after DAP 87 (*H*^2^ of some traits was over 0.7 after DAP 94), indicating the usefulness of the traits for quantitative genetic analyses such as genome-wide association studies (GWAS) and quantitative trait locus (QTL) mapping. On the contrary, *H*^2^ of canopy temperature showed a random pattern. Canopy temperature is expected be useful for genotype selection on certain days but not others. This was probably due to two reasons. First, during periods when cotton plants have adequate soil moisture, there may be little or no temperature difference between drought resistant and non-resistant genotypes. Further, wide row and plant spacing provided more air movement and less competition among plants for water. Second, the experiment was small and might not have used sufficiently large samples to discern statistically significant differences in canopy temperature. Thus, it would be better to study canopy temperature in experiments with irrigation treatments and larger populations. *H*^2^ values of growth rates (dynamic traits) showed an increasing trend until reaching maximal levels (>0.7) in the middle of the growing season (DAP 65 to 72 or DAP 72 to 87). The growth rates’ *H*^2^ over the entire growing season (DAP 51 to 94) were also larger than 0.6. Low-yielding genotypes may continue to grow vegetatively while high-yielding early- and late-season genotypes are in reproductive growth stages during the period from DAP 72 to 87.

## Discussion

Compared with other existing integrated systems, GPhenoVision is a field-based HTP system that consists of multiple high-resolution imaging modalities. Modularity and customizability are two key features of the GPhenoVision system. The modular design is implemented and demonstrated at various levels. The GPhenoVision system is firstly decoupled into four subsystems, with each providing a particular system function: mechanical platform for system mobility, electrical system for powering, and sensing system and software for data acquisition. In particular, the sensing system and data acquisition software are separated as two parts, because the sensing system addresses hardware concerns (e.g. sensor installation position and communication interface) while the data acquisition software provides efficient solutions for user interaction and data management (e.g. data transfer, visualization, and storage). Subsystems are modules in the GPhenoVision system, and replacement/modification of any module will not affect other modules or the entire system functionality as long as the replacement/modification follows the same module interface. For instance, the electrical system is a valid module to the entire system as long as it provides stable 120 V AC power output (the module interface), regardless of which power source (either the tractor secondary alternator or a separate power generator) is being used. Individual subsystems are also decoupled into modules for certain functions, and the DAQ software primarily demonstrates the modular design at this level. The DAQ software uses a multilayered architecture, with each layer being a software module. The synchronization of the sensor control, and data cache and transfer layers provides a mechanism for avoiding data acquisition latency due to the large difference between the data volume generated by imaging sensors and the input/output (I/O) speed of hard drives. Although the data transfer layer with an SSD can achieve a writing speed up to 520 MB/s, which is adequate for most imaging sensors such as LiDARs and hyperspectral cameras, they can be replaced by new hardware and system writing functions to fulfill requirements of higher resolution (thus data volume) sensors without modification of other software modules. The sensor control layer is further decoupled, and each sensor thread is considered as a module in this layer with the module interface of the EFSM. Any EFSM-based sensor thread can be properly controlled by the sensor control layer regardless of the differences in implementation of sensor control. For instance, the RTK-GPS needs a simple LabVIEW function to initialize a serial port for communication/data transfer, whereas the hyperspectral camera needs multiple SDK functions to initialize the SDK library, open a USB3 port for communication/data transfer, and configure camera parameter. Although the initialization implementation of the two sensors is different, the sensor control layer turns them into the same state (sensor initialization) due to the modular design. In other word, sensors (sensor threads) are interchangeable to the sensor control layer because they follow the same module interface. The modularity at various levels does not only simplify the system development and maintenance, but also increases the system customizability. Various sensors can be conveniently integrated into (or removed from) the system for different phenotypes by adding (or deleting) EFSM-based sensor threads in the sensor control layer of the DAQ software. In addition, integrated sensors can be readily selected to use (or not use) in the DAQ software due to the modular design. As the priority of phenotypic traits changes between different growth stages, the system can use various combinations of imaging modules without modification of source code. For instance, canopy temperature becomes less meaningful in late stages such as cotton boll maturity, and thus the thermal camera can be turned off to reduce data storage space. The modular design ensures the stability of customization of the GPhenoVision system in various situations, because turning-off or malfunction of one sensor (module) will not affect the use of other sensors (modules).

The calibration and validation results showed that the RGB-D, thermal, and hyperspectral cameras achieved sufficient measurement performance for measuring various types of phenotypic traits. The RGB-D camera is used to measure morphological traits, and plant height as a representative morphological trait has been repeatedly measured by the RGB-D camera with a difference of 3–4 cm to manual measurements. If considering the field conditions with a wind speed of 3–6.6 m/s, this measurement error is acceptable. The thermal camera is used to measure canopy temperature, and it achieved the nominal measurement accuracy (0.5 °C) in both the laboratory and field conditions. In addition, there was no difference between measurements of plants under shaded and unshaded areas, suggesting no shading effect on the canopy temperature measurement during the regular data collection. The hyperspectral camera provides a high spectral sampling interval of 2.6 nm with a spectral shift less than 1 nm, and the best and worst spatial resolutions are 2.2 mm/pixel and 6.8 mm/pixel, respectively. Given this level of spectral and spatial resolutions, the hyperspectral camera may provide ample information to study plant physiological status (e.g. photosynthesis and diseases) at the organ level, but these need to be further studied by considering experimental design, plant material preparation, agronomic practices, and data analysis.

Although the present study was small-scale, it demonstrated the usefulness of measured phenotypic traits for genomics studies and breeding programs. The sensing capabilities of the GPhenoVision system created new opportunities for measuring multi-dimensional morphological traits such as projected leaf area and canopy volume. In particular, cotton plants continued vegetative growth along different dimensions during the growing season, so it would be better to characterize canopy growth and development using morphological traits in multiple dimensions. Additionally, growth rates were calculated for morphological traits in multiple dimensions, providing the possibility of studying relationships between canopy architecture at different growth stages and plant reproductive efficiency. In contrast, the usefulness of canopy temperature mostly depended on plant growth and environmental conditions. Precipitation was only 0.36 mm/day during DAP 51 to DAP 65, resulting in a large variation of canopy temperature among genotypes on DAP 65. Therefore, different levels of irrigation are necessary to study drought-resistant genotypes by using thermal imaging data.

The GPhenoVision system is modular and customizable, and has demonstrated the potential for genetics/genomics studies and breeding programs, but it should be acknowledged that several parts of the system can be further improved. First, the current illumination configuration (just relying on solar radiation) was not optimal for all three imaging modules. The RGB-D and thermal cameras worked well in the shading condition, whereas the hyperspectral camera showed a relatively low signal intensity due to the reduced incident light. The enclosure can be further split into two sections: RGB-D and thermal section and hyperspectral section. Additional illumination sources can be configured to increase the incident light intensity (and thus reflectance intensity in images) in the section for the hyperspectral camera. Second, challenges still remain in the development of data processing algorithms to take full advantage of the sensing capabilities of the GPhenoVision system. The algorithms in the present study were developed for processing images collected in the SPL field, and need a significant modification for data collected in fields with regular plot layout. In addition, new algorithms should be able to accurately extract complex traits from 3D or hyperspectral images, which require advanced techniques in computer vision and machine learning. For instance, plant components such as flowers or cotton bolls can potentially be detected using convolutional neural networks, even in field conditions containing a complex background. Moreover, processing speed will be an important concern that affects phenotyping throughput. Therefore, it is necessary to consider new computational approaches such as cloud computing and GPU-based optimization to speed up algorithms for trait extraction. In addition, image data are usually in high volume, posing challenges in data storage, management, and sharing. If a project involves researchers from the same institution, a viable solution is to use a storage service maintained by local agencies; otherwise, cloud-based services need to be considered such as ‘CyVerse’, because they can provide reliable data storage, management, and sharing to users in different regions.

## Conclusions

The GPhenoVision system reported in this study can control RGB-D, thermal, and hyperspectral cameras to collect images of cotton plants in field conditions. The proposed sensing system structure and DAQ software architecture would allow rapid development of a custom FB-HTP system that could handle imaging sensors that generate high-volume data. The validation and calibration results showed that the three cameras could provide accurate raw data for phenotyping purposes. Most of the measured traits had *H*^2^ over 0.5 (some over 0.7), confirming the usefulness of using the GPhenoVision system in genomics/genetics studies. Future studies will be focused on developing image processing algorithms to extract more traits and deploying the system in a large-scale experiment for genetic analyses.

## Methods

### Experimental design and field data collection

The GPhenoVision system is intended to be eventually used for experiments involving thousands of lines, so it was necessary to evaluate system capability in a phenotyping scenario. As the entire project was at a fledgling stage, the system was evaluated in a small-scale experiment with a special field layout. A field (33.727458 N, -83.299273 W) contained 132 plants (11 plants per row × 12 rows), and used a single plant layout (SPL) where individual plots had in-row and across-row width of 1.52 m (see Supplementary Figure S4). The ‘SPL’ field was planted on 25 May 2016 with each plot comprising a single plant sampling 23 different genotypes. The genotypes belonged to three groups: elite *Gossypium hirsutum* (*G. hirsutum*), *Gossypium barbadense* (*G. barbadense*, represented by a single elite cultivar), and “exoticâ” *G. hirsutum* genotypes, which include wild and elite cottons not adapted to the study area.

The field was scanned using the system on five days in 2016 including 14 July (51 days after planting, DAP 51), 28 July (DAP 65), 4 August (DAP 72), 19 August (DAP 87), and 26 August (DAP 94). This covered two cotton growth stages: (1) canopy development (DAP 30-80) and (2) flower and boll development (DAP 80-120). No irrigation was scheduled during the data collection period. To obtain daily maximum canopy surface temperature, data collection was strictly conducted in a period from 1200 to 1500 hours on each day (see Supplementary Table S7). Although it could collect data with all imaging modules (see Supplementary Figure S5 for representative images from all imaging modules), the GPhenoVision system in the present study, ran at a constant speed of 1 m/s, with depth, color, and thermal modules in a continuous scanning mode. To save storage space, the operator manually controlled the DAQ software to start/stop saving images at the beginning/end of each row. Cotton fiber was manually harvested and weighed for individual plants on 4 November 2016 (DAP 163). Although a precipitation sensor was not available on the farm during the data collection period, precipitation information was obtained from a public service (Weather Underground, The Weather Company, Atlanta, GA, USA) based on sensors 11 km away from the SPL field.

### Extraction of phenotypic traits

After all plants germinated, positions of individual plants (defined as the position of plant main stem) were surveyed using an RTK-GPS (Cruizer II, Raven Industries Inc., Sioux Falls, SD, USA). Each of the collected images (depth, color, and thermal images) had a corresponding GPS record, and this record indicated the image acquisition position representing the image center. In the configuration (field layout and camera installation position) of the present study, a depth, color, or thermal image captured a single plant with little or no coverage of adjacent plants if that plant was located close to the image center (image acquisition position). Because image acquisition was in a continuous mode, there were multiple images that contained one particular plant. To select only one image from each type of camera for each plant, Euclidean distances were calculated between the physical position of a plant and acquisition positions of all images. The depth, color, and thermal images with the minimum distances to the center of a plant were assigned to that plant for phenotypic trait extraction.

**Figure 8:**
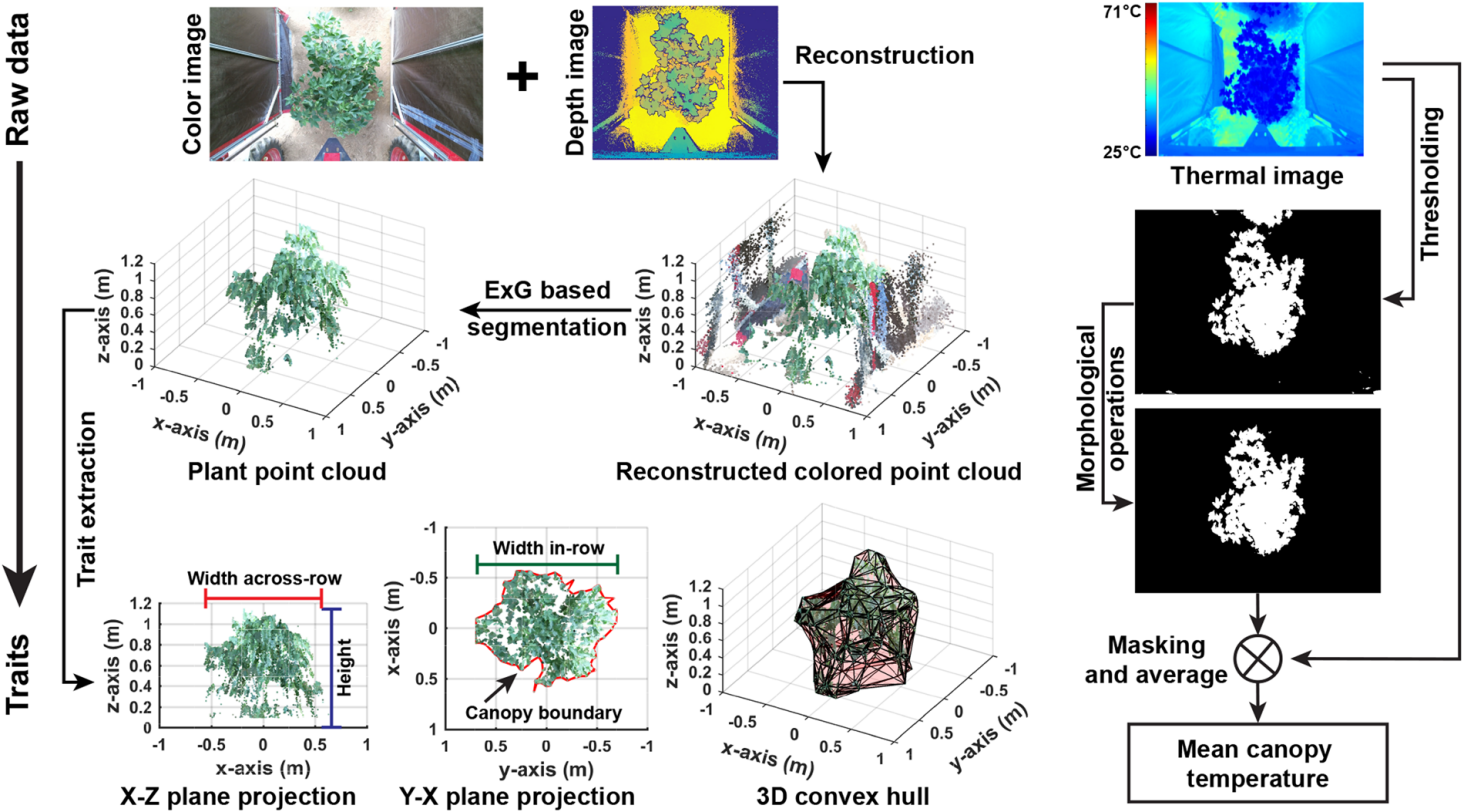
Flowchart of extracting morphological traits from depth and color images, and canopy temperature from thermal images. Excess green (ExG) index was used to segment plants from background in colored point clouds.

Subsequent image processing was performed on each plant’s images to extract phenotypic traits including five morphological traits and canopy temperature. Depth and color images were used to extract the morphological traits (left block in Fig. 8). The raw depth and color images were firstly reconstructed to colored point clouds using a built-in function provided by the Kinect v2 sensor SDK, and a color filter (threshold of 0.15) based on excess green (ExG) index was applied to the colored point clouds to segment canopy points from the background. The plant points were used to calculate the five morphological traits: (1) plant height (H) was the distance between the ground surface (z equals 0) and the highest plant point; (2) width across-row (WAR) was the maximum distance along the x-axis; (3) width in-row (WIR) was the maximum distance along the y-axis; (4) projected leaf area (PLA) was the area covered by the canopy boundary; and (5) canopy volume (CV) was calculated using convex hull algorithm. Growth rates were calculated based on differences in the morphological traits between two measurement days to quantify dynamic changes of plant canopy. In total, growth rate of the morphological traits was calculated in five periods including DAP 51–65, DAP 65-72, DAP 72-87, DAP 87-94, and DAP 51-94.

Canopy temperature was represented by the difference between canopy and air temperature (Tc-Ta). Air temperatures were retrieved from environmental data collected by the auxiliary sensing unit, whereas mean canopy temperatures were calculated from thermal images (right block in Fig. 8). An adaptive thresholding algorithm (Otsu) was performed on each image to segment plants from the ground and the tractor frame. In the resultant binary images, connected components were identified and their centroid and circumcircle diameter were calculated. The connected component in the most central position was recognized as the target plant mask using Equation 3, and connected components representing parts of neighboring plants and/or weeds were removed using Equation 4. The final mask was subsequently applied to the original thermal image to calculate mean canopy temperature.

3 $$Inde{x}_{plant}^{CC}=argmin(\sqrt{{({x}_{i}-{I}_{xcenter})}^{2}+{({y}_{i}-{I}_{ycenter})}^{2}}),\,i=1,2,\ldots ,n$$

where *Index*_*plant*_ is the index of the connected component (CC) in the most central position, *I*_*xcenter*_ and *I*_*ycenter*_ are the x and y coordinates of the image center, and *i* is the index of a given connected component.

4 $$C{C}_{i}^{validated}=(\begin{array}{cc}\mathrm{1,} & ({r}_{i}+{r}_{c})\le \sqrt{{({x}_{i}-{x}_{c})}^{2}+{({y}_{i}-{y}_{c})}^{2}}\\ \mathrm{0,} & {\rm{otherwise}}\end{array},\,i=1,2,\ldots ,n$$

where *CC*^*validated*^ is a flag for a connected component, 1 or 0 means to include/exclude the connected component in the mask, *x*_*i*_, *y*_*i*_, and *r*_*i*_ are the x and y coordinates and the circumcircle diameter of the *i* th connected component, *x*_*c*_, *y*_*c*_, and *r*_*c*_ are the x and y coordinates and the circumcircle dimeter of the target plant connected component.

### Statistical analyses

Pearson correlation analyses were conducted between fiber yield and the extracted traits (or growth rates), and correlation coefficient (*r*) was used as an indicator to evaluate the potential of traits for establishing a yield prediction model useful to select high-yielding genotypes in breeding programs. Analysis of variance (ANOVA) with post-hoc Tukey-Kramer tests were conducted on the extracted traits among three genotype groups, exploring differences in plant growth and development between various cultivated and exotic species. In addition, broad sense heritability (*H*^2^) was calculated for individual traits, and used as an indicator to evaluate the usefulness of a trait for genotype selection and/or quantitative genetic analyses such as genome-wide association studies (GWAS) and quantitative trait locus (QTL) mapping. ANOVA tests were performed on the extracted traits measured on each day between individual *G. hirsutum* genotypes. In the resultant ANOVA tables, the mean sum of squares (MS) for the sources of ‘genotype’ and ‘error’ were the variances due to genotype and environment (including measurement error), respectively. Accordingly, *H*^2^ values were calculated for individual traits using Equation 5 ^30^. The *G. barbadense* species included only one genotype (Pima S6) in the present study. To avoid effects due to various species, data points of the *G. barbadense* species (Pima S6) were excluded from correlation analyses with fiber yield and *H*^2^ calculation. All tests were performed in SAS (SAS 9.4, SAS Institute Inc., Cary, NC, USA) using a significance level of 0.05.

5 $${H}^{2}=\frac{{V}_{G}}{{V}_{P}}=\frac{{V}_{G}}{{V}_{G}+{V}_{E}}$$

where *H*^2^ is the broad sense heritability, *V*_*P*_ is the total phenotypic variance, and *V*_*G*_ and *V*_*E*_ are phenotypic variances due to genotype and environment effects.

## Electronic supplementary material

Supplementary files
