# Supplementary material for: GPhenoVision: A Ground Mobile System with Multi-modal Imaging for Field-Based High Throughput Phenotyping of Cotton
Source: Sci Rep. 2018 Jan 19;8:1213. doi: 10.1038/s41598-018-19142-2 (PMC5775337; doi:10.1038/s41598-018-19142-2)
Supplement: Supplementary file 1 — Supplementary files [file 41598_2018_19142_MOESM1_ESM.doc]

**GPhenoVision: A Ground Mobile System with Multi-modal Imaging for Field-Based High Throughput Phenotyping of Cotton**

Supplementary Materials

Yu Jiang^1^, Changying Li^1^,[[1]](#footnote-2), Jon Robertson^2^, Shangpeng Sun^1^, Rui Xu^1^, and Andrew Paterson^2,3^

^1^ College of Engineering, The University of Georgia, Athens, 30602 Georgia, United States of America

^2^ College of Agricultural & Environmental Sciences, The University of Georgia, Athens, 30602 Georgia, United States of America

^3^ Franklin College of Arts and Sciences, The University of Georgia, Athens, 30602 Georgia, United States of America

**Table S1.** Detailed electrical information for units in the ‘GPhenoVision’ system

| Unit | Device | Voltage | Power consumption |
| --- | --- | --- | --- |
| Primary sensing unit |  |  | 206 W |
|  | Kinect v2 | 12 V | 32 W |
|  | FLIR A655sc | 12 V | 24 W |
|  | Hyperspectral camera | 12 V | 60 W |
|  | Rugged laptop | 19 V | 90 W |
| Auxiliary sensing unit |  |  | 12.5 W |
|  | Microcontroller system | 5 V | 12.5 W |
|  | BME280 sensor | 3 V | Powered by the microcontroller system |
| Positioning unit |  |  | 36 W |
|  | RTK-GPS receiver, console, and field hub | 12 V | 36 W |
| Network router |  |  | 42 W |
|  | Wired router | 12 V | 42 W |
| Total |  |  | 296.5 W |

**Table S2.** Regression analysis results between sensor and manual measurements on different days.

| Date | Regression equation | R^2^ | RMSE (m) | Average wind speed (m/s) |
| --- | --- | --- | --- | --- |
| 28 July 2016 | 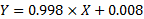 | 0.99 | 0.030 | 6.6 |
| 4 August 2016 | 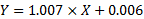 | 0.99 | 0.037 | 5.6 |
| 19 August 2016 | 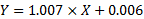 | 0.99 | 0.036 | 3 |

**Table S3.** Precipitation information acquired for different periods in 2016

| Period | Period precipitation (mm) | Accumulated precipitation (mm) | Average daily precipitation (mm) |
| --- | --- | --- | --- |
| 0525 to 0714 (DAP 1 to 51) | 111.76 | 111.76 | 2.23 |
| 0715 to 0728 (DAP 52 to 65) | 5.08 | 116.84 | 0.36 |
| 0729 to 0804 (DAP 66 to 72) | 178.82 | 295.66 | 25.55 |
| 0805 to 0819 (DAP 73 to 87) | 86.87 | 382.53 | 6.21 |
| 0820 to 0826 (DAP 88 to 94) | 22.35 | 404.88 | 3.19 |


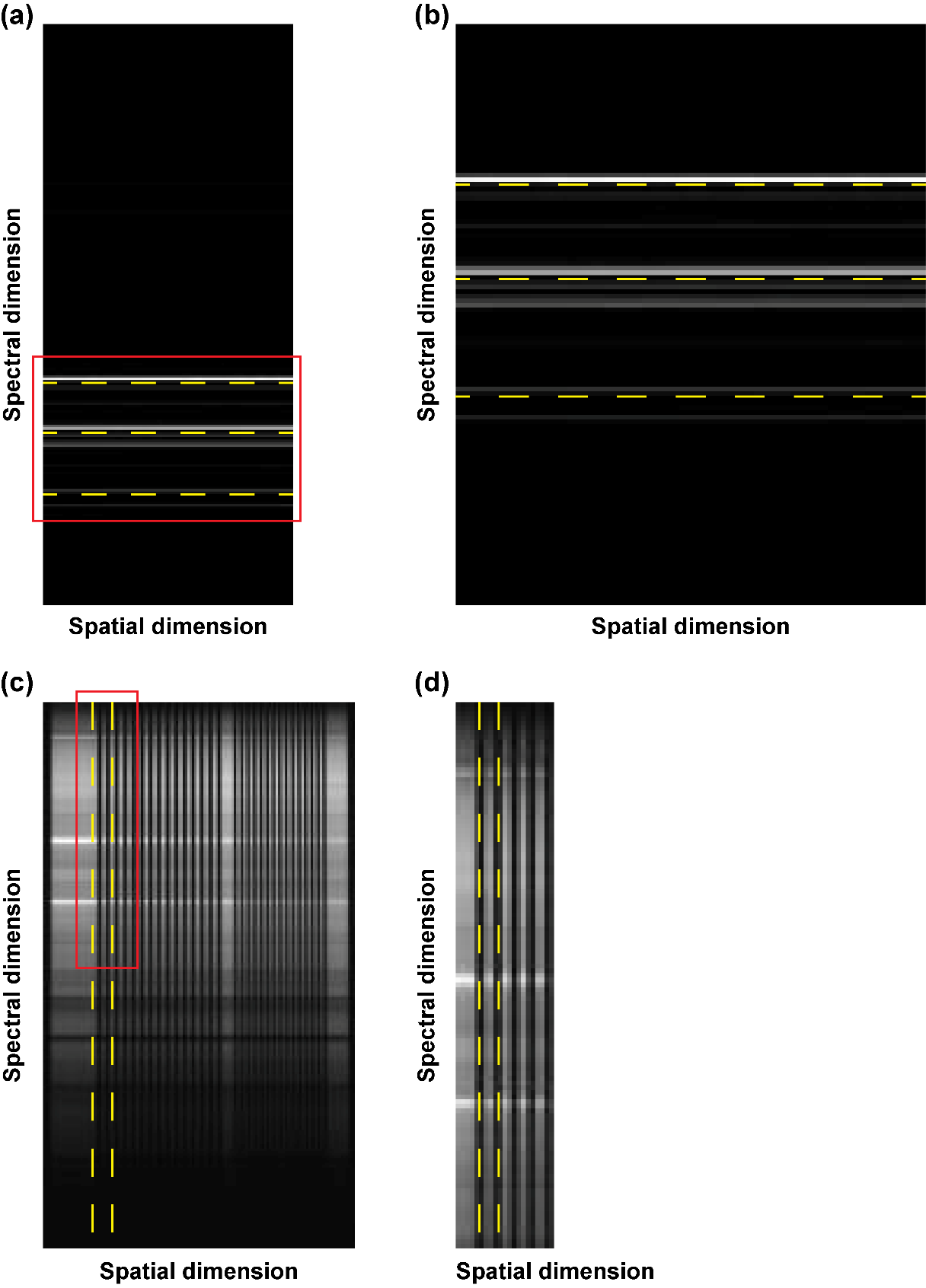


**Figure S1.** Collected images used to evaluate distortion effects of the hyperspectral camera: (a) raw image of one scan line for a Krypton calibration lamp, (b) close-up image of the red rectangle region in (a), (c) raw image of one scan line for a line-pair pattern, and (d) close up image of the red rectangle region in (c). Yellow lines were reference straight lines (either horizontal or vertical).There was no obvious curvature between bright stripes and the reference lines on either the horizontal or vertical direction, indicating no obvious distortion of the hyperspectral camera.

**Figure S2.1.** Correlation matrix between fiber yield and phenotypic traits measured on 14 July 2016 (Day after planting, DAP 51). Yield stands for fiber yield (in g) of individual plants. Phenotypic traits include plant height (H, in m), width in-row (WIR, in m), width across-row (WAR, in m), projected leaf area (PLA, in m^2^), canopy volume (CV, in m^3^), and difference in canopy-air temperature (TCA, also known as Tc-Ta, in °C). Pearson correlation coefficient (r value) is listed in each scattering plot, and red color indicates the correlation is statistically significant.

**Table S4.1.** P-values of Pearson correlation coefficients in Figure S2.1.

| Trait | Yield | H | WIR | WAR | PLA | CV | TcTa |
| --- | --- | --- | --- | --- | --- | --- | --- |
| Yield | 1 | 1.35E-12 | 1.58E-13 | 7.92E-15 | 8.14E-13 | 5.19E-10 | 1.47E-12 |
| H | 1.35E-12 | 1 | 7.83E-27 | 4.06E-31 | 2.08E-27 | 3.80E-29 | 1.65E-13 |
| WIR | 1.58E-13 | 7.83E-27 | 1 | 1.84E-37 | 1.70E-39 | 3.39E-22 | 2.16E-18 |
| WAR | 7.92E-15 | 4.06E-31 | 1.84E-37 | 1 | 7.32E-38 | 4.50E-23 | 2.12E-17 |
| PLA | 8.14E-13 | 2.08E-27 | 1.70E-39 | 7.32E-38 | 1 | 3.22E-34 | 1.43E-13 |
| CV | 5.19E-10 | 3.80E-29 | 3.39E-22 | 4.50E-23 | 3.22E-34 | 1 | 3.32E-10 |
| TcTa | 1.47E-12 | 1.65E-13 | 2.16E-18 | 2.12E-17 | 1.43E-13 | 3.32E-10 | 1 |

**Figure S2.2.** Correlation matrix between fiber yield and phenotypic traits measured on 28 July 2016 (DAP 65). Yield stands for fiber yield (in g) of individual plants. Phenotypic traits include plant height (H, in m), width in-row (WIR, in m), width across-row (WAR, in m), projected leaf area (PLA, in m^2^), canopy volume (CV, in m^3^), and difference in canopy-air temperature (TCA, also known as Tc-Ta, in °C). Pearson correlation coefficient (r value) is listed in each scattering plot, and red color indicates the correlation is statistically significant.

**Table S4.2.** P-values of Pearson correlation coefficients in Figure S2.2.

| Trait | Yield | H | WIR | WAR | PLA | CV | TcTa |
| --- | --- | --- | --- | --- | --- | --- | --- |
| Yield | 1 | 6.74E-14 | 1.50E-12 | 7.04E-11 | 1.93E-10 | 6.34E-07 | 1.01E-05 |
| H | 6.74E-14 | 1 | 8.52E-25 | 4.40E-32 | 1.67E-25 | 3.16E-21 | 2.71E-07 |
| WIR | 1.50E-12 | 8.52E-25 | 1 | 6.30E-29 | 1.33E-40 | 1.19E-21 | 4.57E-08 |
| WAR | 7.04E-11 | 4.40E-32 | 6.30E-29 | 1 | 3.40E-38 | 2.12E-25 | 1.82E-08 |
| PLA | 1.93E-10 | 1.67E-25 | 1.33E-40 | 3.40E-38 | 1 | 7.29E-34 | 2.98E-08 |
| CV | 6.34E-07 | 3.16E-21 | 1.19E-21 | 2.12E-25 | 7.29E-34 | 1 | 3.29E-06 |
| TcTa | 1.01E-05 | 2.71E-07 | 4.57E-08 | 1.82E-08 | 2.98E-08 | 3.29E-06 | 1 |

**Figure S2.3.** Correlation matrix between fiber yield and phenotypic traits measured on 04 August 2016 (DAP 72). Yield stands for fiber yield (in g) of individual plants. Phenotypic traits include plant height (H, in m), width in-row (WIR, in m), width across-row (WAR, in m), projected leaf area (PLA, in m^2^), canopy volume (CV, in m^3^), and difference in canopy-air temperature (TCA, also known as Tc-Ta, in °C). Pearson correlation coefficient (r value) is listed in each scattering plot, and red color indicates the correlation is statistically significant.

**Table S4.3.** P-values of Pearson correlation coefficients in Figure S2.3.

| Trait | Yield | H | WIR | WAR | PLA | CV | TcTa |
| --- | --- | --- | --- | --- | --- | --- | --- |
| Yield | 1 | 9.79E-14 | 4.84E-12 | 4.09E-13 | 1.15E-11 | 4.04E-11 | 2.02E-01 |
| H | 9.79E-14 | 1 | 1.48E-23 | 3.80E-24 | 3.74E-23 | 1.97E-24 | 8.61E-03 |
| WIR | 4.84E-12 | 1.48E-23 | 1 | 1.06E-27 | 1.10E-41 | 3.31E-27 | 1.02E-02 |
| WAR | 4.09E-13 | 3.80E-24 | 1.06E-27 | 1 | 5.46E-36 | 3.22E-22 | 2.05E-01 |
| PLA | 1.15E-11 | 3.74E-23 | 1.10E-41 | 5.46E-36 | 1 | 8.94E-34 | 6.05E-02 |
| CV | 4.04E-11 | 1.97E-24 | 3.31E-27 | 3.22E-22 | 8.94E-34 | 1 | 1.08E-01 |
| TcTa | 2.02E-01 | 8.61E-03 | 1.02E-02 | 2.05E-01 | 6.05E-02 | 1.08E-01 | 1 |

**Figure S2.4.** Correlation matrix between fiber yield and phenotypic traits measured on 19 August 2016 (DAP 87). Yield stands for fiber yield (in g) of individual plants. Phenotypic traits include plant height (H, in m), width in-row (WIR, in m), width across-row (WAR, in m), projected leaf area (PLA, in m^2^), canopy volume (CV, in m^3^), and difference in canopy-air temperature (TCA, also known as Tc-Ta, in °C). Pearson correlation coefficient (r value) is listed in each scattering plot, and red color indicates the correlation is statistically significant.

**Table S4.4.** P-values of Pearson correlation coefficients in Figure S2.4.

| Trait | Yield | H | WIR | WAR | PLA | CV | TcTa |
| --- | --- | --- | --- | --- | --- | --- | --- |
| Yield | 1 | 3.40E-14 | 3.02E-12 | 5.42E-11 | 9.58E-13 | 1.08E-09 | 7.65E-06 |
| H | 3.40E-14 | 1 | 4.48E-19 | 4.86E-18 | 4.47E-21 | 2.66E-20 | 7.54E-04 |
| WIR | 3.02E-12 | 4.48E-19 | 1 | 1.58E-18 | 5.16E-34 | 2.03E-26 | 3.85E-03 |
| WAR | 5.42E-11 | 4.86E-18 | 1.58E-18 | 1 | 1.22E-34 | 3.91E-28 | 8.45E-05 |
| PLA | 9.58E-13 | 4.47E-21 | 5.16E-34 | 1.22E-34 | 1 | 1.77E-42 | 7.95E-04 |
| CV | 1.08E-09 | 2.66E-20 | 2.03E-26 | 3.91E-28 | 1.77E-42 | 1 | 9.02E-04 |
| TcTa | 7.65E-06 | 7.54E-04 | 3.85E-03 | 8.45E-05 | 7.95E-04 | 9.02E-04 | 1 |

**Figure S2.5.** Correlation matrix between fiber yield and phenotypic traits measured on 26 August 2016 (DAP 94). Yield stands for fiber yield (in g) of individual plants. Phenotypic traits include plant height (H, in m), width in-row (WIR, in m), width across-row (WAR, in m), projected leaf area (PLA, in m^2^), canopy volume (CV, in m^3^), and difference in canopy-air temperature (TCA, also known as Tc-Ta, in °C). Pearson correlation coefficient (r value) is listed in each scattering plot, and red color indicates the correlation is statistically significant.

**Table S4.5.** P-values of Pearson correlation coefficients in Figure S2.5.

| Trait | Yield | H | WIR | WAR | PLA | CV | TcTa |
| --- | --- | --- | --- | --- | --- | --- | --- |
| Yield | 1 | 5.97E-09 | 1.56E-10 | 2.14E-12 | 1.36E-10 | 6.20E-08 | 1.58E-02 |
| H | 5.97E-09 | 1 | 8.69E-12 | 3.24E-16 | 5.50E-15 | 1.06E-16 | 9.47E-01 |
| WIR | 1.56E-10 | 8.69E-12 | 1 | 3.20E-17 | 3.81E-35 | 2.10E-18 | 8.53E-01 |
| WAR | 2.14E-12 | 3.24E-16 | 3.20E-17 | 1 | 5.69E-29 | 1.64E-21 | 8.59E-01 |
| PLA | 1.36E-10 | 5.50E-15 | 3.81E-35 | 5.69E-29 | 1 | 4.66E-34 | 8.60E-01 |
| CV | 6.20E-08 | 1.06E-16 | 2.10E-18 | 1.64E-21 | 4.66E-34 | 1 | 8.70E-01 |
| TcTa | 1.58E-02 | 9.47E-01 | 8.53E-01 | 8.59E-01 | 8.60E-01 | 8.70E-01 | 1 |

**Figure S3.1.** Correlation matrix between fiber yield and daily phenotype growth rates calculated between 14 July 2016 (DAP 51) and 28 July 2016 (DAP 65). Yield stands for fiber yield (in g) of individual plants. Daily phenotype growth rates include plant height (H, in m/day), width in-row (WIR, in m/day), width across-row (WAR, in m/day), projected leaf area (PLA, m^2^/day), and canopy volume (CV, in m^3^/day). Pearson correlation coefficient (r value) is listed in each scattering plot, and red color indicates the correlation is statistically significant.

**Table S5.1.** P-values of Pearson correlation coefficients in Figure S3.1.

| Trait | Yield | H | WIR | WAR | PLA | CV |
| --- | --- | --- | --- | --- | --- | --- |
| Yield | 1 | 2.04E-08 | 1.18E-04 | 1.78E-03 | 6.97E-08 | 1.62E-05 |
| H | 2.04E-08 | 1 | 2.44E-06 | 1.48E-10 | 2.82E-14 | 1.49E-12 |
| WIR | 1.18E-04 | 2.44E-06 | 1 | 6.72E-09 | 4.33E-20 | 1.16E-12 |
| WAR | 1.78E-03 | 1.48E-10 | 6.72E-09 | 1 | 3.49E-20 | 8.37E-14 |
| PLA | 6.97E-08 | 2.82E-14 | 4.33E-20 | 3.49E-20 | 1 | 2.53E-28 |
| CV | 1.62E-05 | 1.49E-12 | 1.16E-12 | 8.37E-14 | 2.53E-28 | 1 |

**Figure S3.2.** Correlation matrix between fiber yield and daily phenotype growth rates calculated between 28 July 2016 (DAP 65) and 04 August 2016 (DAP 72). Yield stands for fiber yield (in g) of individual plants. Daily phenotype growth rates include plant height (H, in m/day), width in-row (WIR, in m/day), width across-row (WAR, in m/day), projected leaf area (PLA, m^2^/day), and canopy volume (CV, in m^3^/day). Pearson correlation coefficient (r value) is listed in each scattering plot, and red color indicates the correlation is statistically significant.

**Table S5.2.** P-values of Pearson correlation coefficients in Figure S3.2.

| Trait | Yield | H | WIR | WAR | PLA | CV |
| --- | --- | --- | --- | --- | --- | --- |
| Yield | 1 | 2.25E-01 | 1.55E-01 | 1.69E-01 | 2.10E-07 | 1.43E-09 |
| H | 2.25E-01 | 1 | 3.55E-02 | 1.98E-02 | 1.55E-02 | 3.76E-03 |
| WIR | 1.55E-01 | 3.55E-02 | 1 | 3.15E-02 | 3.74E-08 | 3.87E-04 |
| WAR | 1.69E-01 | 1.98E-02 | 3.15E-02 | 1 | 3.64E-04 | 2.13E-01 |
| PLA | 2.10E-07 | 1.55E-02 | 3.74E-08 | 3.64E-04 | 1 | 1.37E-12 |
| CV | 1.43E-09 | 3.76E-03 | 3.87E-04 | 2.13E-01 | 1.37E-12 | 1 |

**Figure S3.3.** Correlation matrix between fiber yield and daily phenotype growth rates calculated between 04 August 2016 (DAP 72) and 19 August 2016 (DAP 87). Yield stands for fiber yield (in g) of individual plants. Daily phenotype growth rates include plant height (H, in m/day), width in-row (WIR, in m/day), width across-row (WAR, in m/day), projected leaf area (PLA, m^2^/day), and canopy volume (CV, in m^3^/day). Pearson correlation coefficient (r value) is listed in each scattering plot, and red color indicates the correlation is statistically significant.

**Table S5.3.** P-values of Pearson correlation coefficients in Figure S3.3.

| Trait | Yield | H | WIR | WAR | PLA | CV |
| --- | --- | --- | --- | --- | --- | --- |
| Yield | 1 | 7.07E-01 | 3.39E-02 | 4.19E-02 | 7.82E-08 | 1.78E-04 |
| H | 7.07E-01 | 1 | 1.79E-08 | 6.34E-04 | 4.18E-03 | 7.25E-04 |
| WIR | 3.39E-02 | 1.79E-08 | 1 | 3.76E-07 | 7.03E-14 | 1.52E-09 |
| WAR | 4.19E-02 | 6.34E-04 | 3.76E-07 | 1 | 3.14E-17 | 4.03E-11 |
| PLA | 7.82E-08 | 4.18E-03 | 7.03E-14 | 3.14E-17 | 1 | 3.86E-24 |
| CV | 1.78E-04 | 7.25E-04 | 1.52E-09 | 4.03E-11 | 3.86E-24 | 1 |

**Figure S3.4.** Correlation matrix between fiber yield and daily phenotype growth rates calculated between 19 August 2016 (DAP 87) and 26 August 2016 (DAP 94). Yield stands for fiber yield (in g) of individual plants. Daily phenotype growth rates include plant height (H, in m/day), width in-row (WIR, in m/day), width across-row (WAR, in m/day), projected leaf area (PLA, m^2^/day), and canopy volume (CV, in m^3^/day). Pearson correlation coefficient (r value) is listed in each scattering plot, and red color indicates the correlation is statistically significant.

**Table S5.4.** P-values of Pearson correlation coefficients in Figure S3.4.

| Trait | Yield | H | WIR | WAR | PLA | CV |
| --- | --- | --- | --- | --- | --- | --- |
| Yield | 1 | 4.16E-04 | 6.99E-01 | 2.94E-01 | 3.05E-01 | 8.48E-01 |
| H | 4.16E-04 | 1 | 7.64E-06 | 2.94E-06 | 9.20E-09 | 9.69E-06 |
| WIR | 6.99E-01 | 7.64E-06 | 1 | 4.63E-03 | 3.06E-10 | 3.32E-03 |
| WAR | 2.94E-01 | 2.94E-06 | 4.63E-03 | 1 | 2.12E-14 | 3.30E-08 |
| PLA | 3.05E-01 | 9.20E-09 | 3.06E-10 | 2.12E-14 | 1 | 2.11E-10 |
| CV | 8.48E-01 | 9.69E-06 | 3.32E-03 | 3.30E-08 | 2.11E-10 | 1 |

**Figure S3.5.** Correlation matrix between fiber yield and daily phenotype growth rates calculated between 14 July 2016 (DAP 51) and 26 August 2016 (DAP 94). Yield stands for fiber yield (in g) of individual plants. Daily phenotype growth rates include plant height (H, in m/day), width in-row (WIR, in m/day), width across-row (WAR, in m/day), projected leaf area (PLA, m^2^/day), and canopy volume (CV, in m^3^/day). Pearson correlation coefficient (r value) is listed in each scattering plot, and red color indicates the correlation is statistically significant.

**Table S5.5.** P-values of Pearson correlation coefficients in Figure S3.5.

| Trait | Yield | H | WIR | WAR | PLA | CV |
| --- | --- | --- | --- | --- | --- | --- |
| Yield | 1 | 5.13E-01 | 9.24E-04 | 4.99E-03 | 1.27E-08 | 3.44E-07 |
| H | 5.13E-01 | 1 | 1.63E-07 | 2.19E-11 | 1.83E-07 | 1.34E-07 |
| WIR | 9.24E-04 | 1.63E-07 | 1 | 9.61E-12 | 8.53E-25 | 8.14E-13 |
| WAR | 4.99E-03 | 2.19E-11 | 9.61E-12 | 1 | 2.76E-17 | 1.62E-13 |
| PLA | 1.27E-08 | 1.83E-07 | 8.53E-25 | 2.76E-17 | 1 | 1.22E-32 |
| CV | 3.44E-07 | 1.34E-07 | 8.14E-13 | 1.62E-13 | 1.22E-32 | 1 |

**Table S6.** P-values of ANOVA with post hoc Tukey-Kramer tests for extracted traits among three genotype groups (N*_G.hirsutum_* = 45, N*_G.barbadense_* = 6, N*_exotics_* = 49)

| Trait | *G. hirsutum* | *G. barbadense* | Exotics | p-value |
| --- | --- | --- | --- | --- |
| HeightDAP51 | A | AB | B | <0.0001 |
| HeightDAP65 | A | AB | B | 0.0491 |
| HeightDAP72 | A | B | B | 0.0066 |
| HeightDAP87 | A | C | B | <0.0001 |
| HeightDAP94 | A | B | A | <0.0001 |
| WIRDAP51 | A | A | B | <0.0001 |
| WIRDAP65 | A | A | A | 0.2553 |
| WIRDAP72 | A | A | A | 0.1473 |
| WIRDAP87 | A | B | A | 0.0045 |
| WIRDAP94 | A | B | A | 0.0007 |
| WARDAP51 | A | A | B | 0.0008 |
| WARDAP65 | A | A | A | 0.1338 |
| WARDAP72 | A | B | AB | 0.0304 |
| WARDAP87 | A | B | A | <0.0001 |
| WARDAP94 | A | B | A | <0.0001 |
| PLADAP51 | A | A | B | 0.0008 |
| PLADAP65 | A | A | A | 0.373 |
| PLADAP72 | A | A | A | 0.0912 |
| PLADAP87 | A | B | A | 0.0007 |
| PLADAP94 | A | B | A | 0.0012 |
| VolumeDAP51 | A | A | B | 0.0122 |
| VolumeDAP65 | A | A | A | 0.2281 |
| VolumeDAP72 | A | A | A | 0.1432 |
| VolumeDAP87 | A | B | A | 0.0007 |
| VolumeDAP94 | A | B | A | 0.0006 |
| TcTaDAP51 | B | B | A | 0.0488 |
| TcTaDAP65 | B | A | B | 0.007 |
| TcTaDAP72 | A | A | A | 0.732 |
| TcTaDAP87 | C | A | B | <0.0001 |
| TcTaDAP94 | B | AB | A | 0.0135 |
| 'HeightP1' | A | A | A | 0.1538 |
| 'HeightP2' | A | B | A | 0.0026 |
| 'HeightP3' | A | B | A | <0.0001 |
| 'HeightP4' | A | B | A | 0.0236 |
| 'HeightAll' | A | B | A | <0.0001 |
| 'WIRP1' | A | A | A | 0.2137 |
| 'WIRP2' | A | A | A | 0.1127 |
| 'WIRP3' | B | C | A | 0.0026 |
| 'WIRP4' | A | A | A | 0.1351 |
| WIRAll' | A | B | A | 0.0002 |
| 'WARP1' | A | B | A | 0.0058 |
| 'WARP2' | A | A | A | 0.2620 |
| 'WARP3' | A | B | A | <0.0001 |
| 'WARP4' | A | A | A | 0.5338 |
| 'WARAll' | A | B | A | <0.0001 |
| 'PLAP1' | A | A | A | 0.2375 |
| 'PLAP2' | A | B | AB | 0.0230 |
| 'PLAP3' | A | B | A | <0.0001 |
| 'PLAP4' | A | A | A | 0.7113 |
| 'PLAAll' | A | B | A | 0.0008 |
| 'VolumeP1' | A | A | A | 0.1595 |
| 'VolumeP2' | A | A | A | 0.2499 |
| 'VolumeP3' | A | B | A | 0.0003 |
| 'VolumeP4' | A | A | A | 0.6233 |
| 'VolumeAll' | A | B | A | 0.0006 |


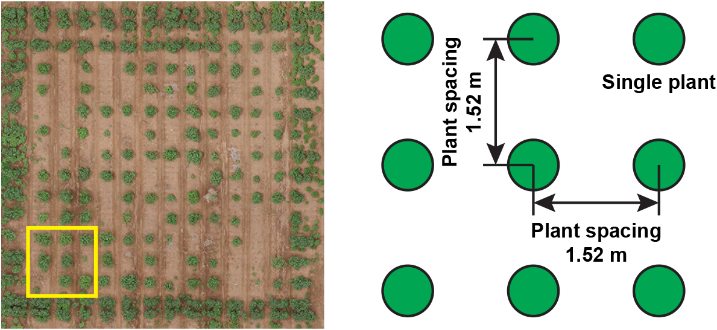


**Figure S4.** The single plant layout (SPL) field used for evaluation of the GPhenoVision system. Individual plants were spaced 1.52 m with each other in the field.

**Table S7.** Time and weather information for data collection conducted in SPL filed on five days in 2016.

| Date | Data collection time | Mean air temperature (°C) | Relative humidity (%) |
| --- | --- | --- | --- |
| 0714 (DAP 51) | 1:46 pm to 2:05 pm | 35.3 | 40 |
| 0728 (DAP 65) | 12:26 pm to 12:42 pm | 32.4 | 48 |
| 0804 (DAP 72) | 12:28 pm to 12:42 pm | 34.5 | 53 |
| 0819 (DAP 87) | 1:14 pm to 1:28 pm | 34.5 | 53 |
| 0826 (DAP 94) | 1:12 pm to 1:29 pm | 36 | 42 |


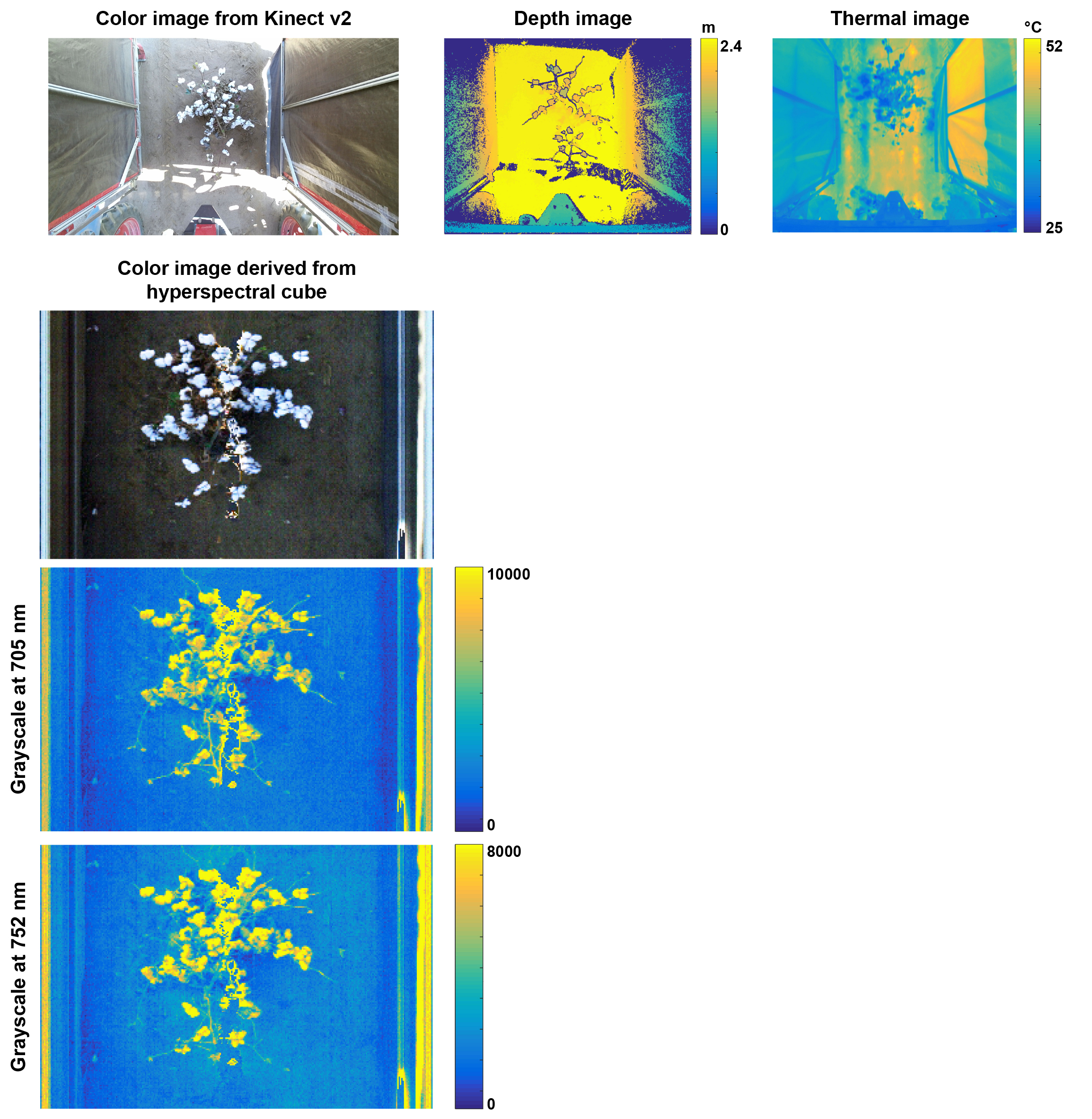


**Figure S5.** Representative images collected on 1 Nov 2016 (160 days after planting, DAP) by the GPhenoVision system with all imaging modules, including color, depth, thermal, and hyperspectral images. One of the color image was derived from the hyperspectral cube with blue, green, and red channels at 473 nm, 570 nm, 649 nm, and two grayscale images at 705 nm and 752 nm.

1. Corresponding author at 712F Boyd Graduate Studies, 200 D. W. Brooks Drive, University of Georgia, Athens, Georgia, 30602, United States of America. Phone: (706) 542‐4696; Fax: (706) 542‐2475; Email: cyli@uga.edu; Website: http://sensinglab.engr.uga.edu/ [↑](#footnote-ref-2)
